# Computational formulation of a multiepitope vaccine unveils an exceptional prophylactic candidate against Merkel cell polyomavirus

**DOI:** 10.3389/fimmu.2023.1160260

**Published:** 2023-06-27

**Authors:** Raihan Rahman Imon, Abdus Samad, Rahat Alam, Ahad Amer Alsaiari, Md. Enamul Kabir Talukder, Mazen Almehmadi, Foysal Ahammad, Farhan Mohammad

**Affiliations:** ^1^ Laboratory of Computational Biology, Biological Solution Centre (BioSol Centre), Jashore, Bangladesh; ^2^ Department of Genetic Engineering and Biotechnology, Jashore University of Science and Technology, Jashore, Bangladesh; ^3^ Clinical Laboratories Science Department, College of Applied Medical Science, Taif University, Taif, Saudi Arabia; ^4^ Division of Biological and Biomedical Sciences (BBS), College of Health and Life Sciences (CHLS), Hamad Bin Khalifa University (HBKU), Doha, Qatar

**Keywords:** Merkel cell polyomavirus (MCV), Merkel cell carcinomas (MCC), immunoinformatics, vaccine design, multiepitope vaccine, molecular dynamics simulation (MD), molecular docking

## Abstract

Merkel cell carcinoma (MCC) is a rare neuroendocrine skin malignancy caused by human Merkel cell polyomavirus (MCV), leading to the most aggressive skin cancer in humans. MCV has been identified in approximately 43%–100% of MCC cases, contributing to the highly aggressive nature of primary cutaneous carcinoma and leading to a notable mortality rate. Currently, no existing vaccines or drug candidates have shown efficacy in addressing the ailment caused by this specific pathogen. Therefore, this study aimed to design a novel multiepitope vaccine candidate against the virus using integrated immunoinformatics and vaccinomics approaches. Initially, the highest antigenic, immunogenic, and non-allergenic epitopes of cytotoxic T lymphocytes, helper T lymphocytes, and linear B lymphocytes corresponding to the virus whole protein sequences were identified and retrieved for vaccine construction. Subsequently, the selected epitopes were linked with appropriate linkers and added an adjuvant in front of the construct to enhance the immunogenicity of the vaccine candidates. Additionally, molecular docking and dynamics simulations identified strong and stable binding interactions between vaccine candidates and human Toll-like receptor 4. Furthermore, computer-aided immune simulation found the real-life-like immune response of vaccine candidates upon administration to the human body. Finally, codon optimization was conducted on the vaccine candidates to facilitate the *in silico* cloning of the vaccine into the pET28+(a) cloning vector. In conclusion, the vaccine candidate developed in this study is anticipated to augment the immune response in humans and effectively combat the virus. Nevertheless, it is imperative to conduct *in vitro* and *in vivo* assays to evaluate the efficacy of these vaccine candidates thoroughly. These evaluations will provide critical insights into the vaccine’s effectiveness and potential for further development.

## Graphical Abstract

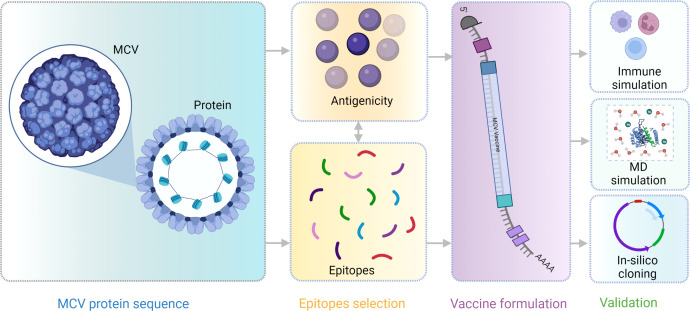

## 1 Introduction

Merkel cell polyomavirus (MCV) is one of the seven currently known human oncoviruses in the human polyomaviruses (HPV) family. It has drawn massive attention due to its link to rare human cancer. The virus induces cancer in its natural host and is a primary agent known to cause Merkel cell carcinoma (MCC) (1, 2). MCV is a causative agent in approximately 43%–100% of MCCs, leading to more incidences in aged and immunocompromised patients (3). MCC, which is an aggressive type of skin cancer, was first described by Cyril Toker in 1972 (1, 4). He discovered that the development of neuroendocrine carcinoma of the skin, also referred to as a “trabecular tumor of the skin,” is associated with MCV infection. The viral infection triggers an abnormal increase in Merkel cells (MCs) and skin mechanoreceptor cells, leading to uncontrolled proliferation (5). The MCs are found deep in the epidermis of the top layer of the skin as innervated clusters of cells close to the nerve endings receiving touch and pressure sensations (6). The MCC is considered the second deadliest form of skin cancer after malignant melanoma, with a mortality rate of 35% (7). Skin cancer ranks as the 17th most prevalent cancer globally and one of the most diagnosed cancers worldwide. In the United States alone, an estimated 9,500 new cases of skin cancer are diagnosed daily (8). Particularly, MCC contributes to approximately 700 annual fatalities (9). However, the etiology and pathogenesis of MCC remain elusive (10, 11).

MCV is a small, circular, non-enveloped, double-stranded DNA virus highly prevalent in humans and causes skin malignancy (12). The virus is classified within the ortho-polyomaviruses family, which encompasses various mammalian polyomaviruses, including simian virus (SV40), murine polyomavirus, and the human BK polyomaviruses and John Cunningham virus (13–16). The prototype genomic sequence of MCV encodes characteristic polyomavirus proteins from opposite strands, including early genes encoding large T antigen and small T antigen and late genes encoding viral capsid proteins (VP1, VP2, and VP3 genes) (11, 17). MCV viral T antigens are oncoproteins expressed in human MCC tumors (18). The oncoproteins, namely, large and small T, play a pivotal role in the transformation of normal cells into cancer cells. They exert their influence by activating tumor suppressor proteins, contributing to the development and progression of cancer (19). The viral proteins 1, 2, and 3 (VP1, VP2, and VP3) are expressed by the virus through three open reading frames, functioning as capsid proteins. The VP1 protein constitutes 70% of the total virus protein particles, and the protein is a major immunogenic component found in the host immune system required for producing pseudo virions (20). MCV causes abnormalities in the skin’s MCs and transforms normal cells into cancer cells. In MCC tumors, VP1 is the major viral protein required to form viral particles and to bind to the site for infection. Anti-VP1 antibodies in the blood indicate chronic disease with MCV (21). Vaccines have successfully been developed against HPV and HBV, targeting the different structural proteins of the viruses (22, 23). The limited understanding of MCC etiology has prevented us from achieving similar successes for MCV, necessitating exploring innovative approaches and treatments for MCC (24). Developing a therapeutic vaccine can be considered a success for the disease that may provide support to enhance the activity of cancer-specific T cells and promote antitumor immunity. The therapeutic vaccines will enhance cellular response by activating antigen-specific CD8+ T cells of patients with MCC-positive tumors.

This study aims to design an efficient multiepitope vaccine against MCV using computational immunoinformatics approaches to provide novel treatment options for MCC. The multiepitope vaccine will generate a more robust immune response to viral particles and peptides (25, 26). It will produce fewer fatal consequences than vaccines developed using complete viral proteins and peptides (27, 28). As MCV T antigens are tumor suppressors in the human body and form cancer cells by altering typical MCs, this newly developed vaccine may help prevent the transformation of MCs to cancer cells in human skin (29). We have designed a vaccine candidate against MCV that binds to MC’s receptor site and can potentially fight against MCV in the human body. Previously, DNA vaccines were developed targeting large and small T antigens or VP1. They produced antitumor effects by inducing cytotoxic and helper T lymphocyte (CTL and HTL) responses in mice (30–32). In this study, we have used selected epitopes of capsid proteins (VP1-VP3) and large and small T antigens and designed multiple epitope vaccine candidates, showing computationally more robust immune responses. However, further *in vitro* and *in vivo* experiments must be conducted to confirm the efficacy of the designed multiepitope vaccines produced using predicted epitopes.

## 2 Materials and methods

**Figure 1:**
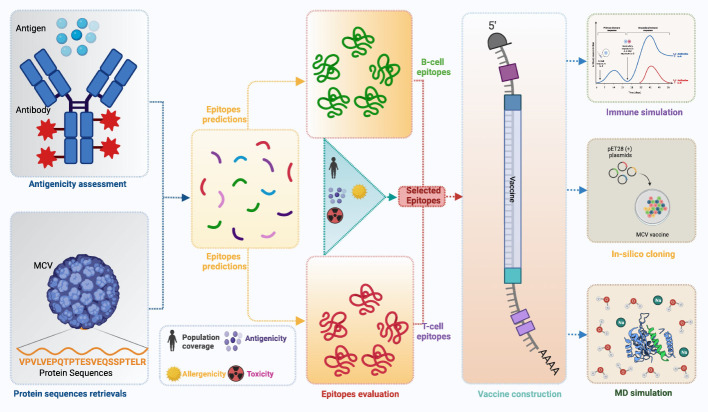
This schematic diagram illustrates the comprehensive workflow employed in the current study for computational multiepitope vaccine design against Merkel cell polyomavirus (MCV). The workflow utilized vital steps, including target antigen identification, epitope prediction, epitope selection, design of multiepitope constructs, structural modeling and validation, *in silico* cloning, and *in silico* evaluation of immunogenicity and efficacy. These steps collectively contribute to developing an optimized multiepitope vaccine candidate for MCV.

A flow chart of the overall procedure applied in these studies, initiated from antigenic protein selection to vaccine construction and evaluation, is illustrated in **Figure 1**.

Also, we have provided detailed information about the servers used in the design of MCV vaccine candidates in **Table 1**. It includes their functions, parameters, and thresholds, which are crucial for predicting antigenicity, epitopes, protein structures, and optimized vaccine design. **Table 1** indicates the specific parameters and thresholds each server uses during its prediction processes. This valuable information will serve as a comprehensive reference guide, highlighting essential servers, their functionality, and the parameters and thresholds used during the computational-based design process of MCV vaccine candidates.

**Table 1:** This table provides a comprehensive compilation of servers employed in the design of Merkel cell polyomavirus (MCV) vaccine candidates, including their functions, parameters, and thresholds.

Server Name	Function	Parameters	Threshold	Reference
UniProt	Protein sequence retrieval	–	–	(33)
VaxiJen v2.0	Antigenicity prediction	Default	0.5	(34)
ANTIGENpro	Antigenicity prediction	Default	–	(35)
NetCTL 1.2	CTL epitope predictions	Default	0.75	(36)
AllergenFP v.1.0	Allergenicity prediction	Default	–	(37)
ToxinPred	Toxicity prediction	Default	0.5	(38)
MHC-I immunogenicity	Immunogenicity prediction	Default	–	(39)
Immune epitope database and analysis resource (IEDB)	T-cell epitope prediction	Default	–	(40)
IFNepitope	IFN-γ inducing epitope prediction	Default	–	(41)
BepiPred 2.0	B-cell epitope prediction	Default	0.5	(42)
ProtParam	Physicochemical property prediction of a protein	Default	–	(43)
SOLpro	Solubility	Default	–	(44)
I-TASSER	Protein structure prediction	–	–	(45)
GalaxyRefine	Refinement and optimization of protein structures	Default	–	(46)
ProSA	Validation of protein structures	Default	–	(47)
Protein Data Bank (PDB)	Experimentally determined 3D protein structures	–	–	(48)
ClusPro 2.0	Protein–protein docking	Default	–	(49)
JCat	Optimizing codon	Default	–	(50)

The servers in the table perform various tasks related to antigenicity prediction, epitope prediction, protein structure prediction, refinement, and optimization.

### 2.1 Proteome retrieval and antigenicity prediction

We obtained the protein sequences of MCV from the UniProt website, a widely accessible database of experimentally characterized protein sequences. UniProt offers comprehensive information regarding these protein sequences, facilitating our research and analysis (51). Five protein sequences, including large T antigen, small T antigen, VP1, VP2, and VP3 of the MCV, were retrieved from the UniProt (Proteome ID: UP000154903). All protein sequences were downloaded in the FASTA file format and submitted to the VaxiJen v2.0 server for antigenicity prediction (34). We utilized a web-based tool to align independent protein sequences, enabling the identification of antigens that exhibited performance based on auto cross-covariance transformation and aiding in the determination of uniform vectors of equal lengths. We selected the proteins with the highest antigenicity for subsequent analysis. The threshold value was 0.5 to predict 12 MHC supertypes, including supertypes A26 and B39 of MHC. Additionally, ANTIGENpro was also used to indicate the antigenicity of the selected proteins (35).

### 2.2 Epitope identification

#### 2.2.1 Cytotoxic T lymphocyte epitope evaluation and selection

We submitted the selected antigenic proteins with the highest antigenicity scores to the NetCTL 1.2 server for CTL epitope prediction, which has a higher predictive capability and sensitivity than other available methods (36, 52). We analyzed CTL epitopes within the 12 HLA-I supertypes (A1, A2, A3, A24, A26, B7, B8, B27, B39, B44, B58, and B62) to select specific antigenic proteins. The proteins with the highest NetCTL scores were selected. A default NetCTL value of 0.75 was used as a cut-off to predict and select a CTL epitope (53). This method combines the prediction of peptide major histocompatibility (MHC) class I binding, proteasomal C_terminal cleavage, and transporter associated with antigen processing (TAP_) transport (54). We evaluated selected CTL epitopes for immunogenic, antigenic, allergenic, and toxicity properties. The immunogenic response of CTLs is the main requirement for vaccine construction. First, the selected epitopes were submitted to the MHC-I immunogenicity tool of the IEDB website to evaluate immunogenic properties (40). Second, selected epitopes were analyzed using the VaxiJen 2.0 server for antigenic evaluation (34). Third, the allergenicity of the selected epitopes was predicted using the AllergenFP v.1.0 server for CTL epitope evaluation (37). Finally, the toxicity of CTL epitopes was evaluated using the ToxinPred server (38). In most cases, we utilized the default parameters of the server for epitope evaluations. In this study, we chose CTL epitopes with immunogenic, antigenic, non-allergenic, and non-toxic properties for the final vaccine constructs.

#### 2.2.3 Helper T lymphocyte epitope evaluation and selection

Helper T lymphocyte (HTL) cells play a crucial role in adaptive immunity, stimulating both humoral and cellular immune responses against foreign antigens (53). To identify HTL epitopes of the MCV protein, we utilized the MHC-II binding allele-IEDB Analysis Resource website as a resource in this study (55). We used the consensus method of 5% percentile for HTL epitope prediction and selection, and 15-mer peptide epitopes were selected. Subsequently, the chosen HTL epitopes were evaluated based on interferon-gamma, interleukin-4, interleukin-10, and antigenicity properties. The interferon-gamma is a type of cytokine critical to both innate and adaptive immunity that plays an essential role in vaccine construction. First, selected epitopes were submitted to the IFNepitope server for interferon-gamma secretion property analysis, which utilized a hybrid method [support vector machine (SVM) and motif method] to analyze the properties (41). Second, the interleukin-4- and interleukin-10-producing ability of the HTL epitopes were predicted by using the IL4Pred and IL10Pred servers (56, 57). Based on the induction and non-induction properties, interleukin-4 and interleukin-10 were selected. Finally, we analyzed the antigenic properties of the HTL epitopes using the VaxiJen 2.0 server (34). The HTL epitopes selected based on the induction ability of interferon-gamma, interleukin-4, and interleukin-10 and antigenicity properties were used for the vaccine constructs.

#### 2.2.3 B-Cell lymphocyte epitope evaluation and selection

Linear B-cell epitopes are crucial in antibody production and the construction of peptide-based vaccines (58). To identify linear B-cell epitopes, we submitted the selected antigenic proteins to the BepiPred 2.0 web tool (42, 59). This tool successfully identified 12-mer peptide epitopes corresponding to the MCV protein. The threshold parameter that has been set was 0.5. The selected B-Cell lymphocyte (BCL) epitopes were further evaluated based on antigenicity, allergenicity, and toxicity properties. Finally, the best BCL epitopes with the highest antigenicity, non-allergenicity, and non-toxicity properties were selected for vaccine construction.

### 2.3 Estimation of population coverage

The distribution of human leukocyte antigen (HLA) alleles and their expression patterns vary country by country and worldwide according to the differences in genomic regions and ethnicities (60). In computational vaccine design, population coverage directly indicates the worldwide effectiveness of the vaccine by evaluating the prevalence of HLA alleles related to the epitope of interest. Therefore, the population coverage was calculated using the T-cell epitopes with their respective HLA-binding alleles. To achieve this, we submitted the selected epitopes along with their allelic information to the IEDB population coverage tool. This tool allows for assessing the coverage provided by the selected epitopes across different populations (61). Population coverage scores were calculated using the HLA hit score derived from the relative allele frequency at a specific locus within a particular population.

### 2.4 Formulation of multiepitope vaccine

The multiepitope vaccine candidate was formulated by properly utilizing previously selected CTL, HTL, and LBL epitopes initiated with a suitable adjuvant linked by a different linker, including EAAAK, AAY, GPGPG, and KK. In this study, the adjuvant, linker, and epitope of the protein were ordered in a way that can elicit maximum immune cell–specific responses and confer protection against the virus (53, 62). However, the epitopes of the vaccines were shuffled and appointed in a different order. Based on the antigenicity and physiochemical properties, the best confirmation was selected for further evaluation. Initially, an ideal adjuvant receptor was identified through an advanced literature search to enhance the immunogenicity of MCV protein fuse with Fc of human IgG. It has been found that TLR agonists TLR 2, 4, 5, 7, and 9 play an essential role in the pattern recognition of MCV protein (63). However, in this study, the TLR4 agonist was used as an adjuvant due to the maximal rate of synthesis ability and activating the highest immune responses against the MCV (64). The TLR4 agonist known as 50S ribosomal protein L7/L12 of *Mycobacterium tuberculosis* was retrieved from the UniProtKB (ID: P9WHE3) and used as the adjuvant to enhance the immunogenicity of the vaccine candidate (65). Specific linker molecules were employed to fuse the peptide sequences in the study. The front of the adjuvant was attached with a bifunctional linker EAAAK. Subsequently, CTL, HTL, and LBL epitopes were linked together through AAY, GPGPG, and KK linkers, respectively (66). Initially, the vaccine adjuvant was attached to the front of the vaccine using the EAAAK linker, which consists of helix-forming peptides of various lengths. This linker serves to separate the two weakly interacting β-domains (67). On the other hand, selected CTL was linked using Ala-Ala-Tyr (AAY) linkers, while HTL was linked with Gly-Pro-Gly-Pro-Gly (GPGPG) linkers. In addition, LBL has linked to Lys-Lys (KK) linkers (53). The AAY linker, which is a cleavage site for the proteasome, was used to affect protein stability, reduce immunoreactivity, and enhance epitope presentation. The GPGPG linker, known as the glycine–proline linker, prevents the formation of “junctional epitopes” and facilitates the immunological process (68). In addition, the bi-lysine KK linker helps preserve independent immunological activities during the vaccine formulation (69). The peptides of the construct were fused with each other using the selected linker due to their ability to provide support for structure flexibility, improve protein stability, and play an important role in increasing the biological activity of the vaccine construct (53, 70).

### 2.5 Physicochemical and immunological properties analysis

The efficacy of the vaccine candidate was assessed by evaluating its physiological, antigenic, immunogenic, allergenic, and soluble properties. The physicochemical properties of the vaccine construct were analyzed using the ProtParam tool, enabling a comprehensive examination of its characteristics (43).. The tool calculated the physiological properties, including molecular weight, theoretical pi, and the number of positively charged residues (Arg + Lys). The chemical formula of the vaccine, the whole number of atoms, coefficient extinction, *in vitro* and *in vivo*, half-life, instability, aliphatic index, and grand average of hydropathicity (GRAVY) value were also determined by using the tool. We evaluated the antigenic, immunogenic, and allergenic properties of the vaccine constructs by utilizing specific web tools. The VaxiJen 2.0 tool was employed to assess antigenicity, the MHC-I immunogenicity tool from the IEDB was used to determine immunogenicity, and the AllergenFP v.1.0 web tool was utilized to evaluate allergenicity. These analyses provided valuable insights into the properties of the vaccine constructs (34, 37, 55). The solubility of the vaccine construct was also evaluated with the help of the SOLpro web-based tool (44).

### 2.6 Vaccine structure prediction, refinement, and validation

#### 2.6.1 Secondary structure prediction

For analyzing the extended strand, alpha-helix, and random coils for the secondary structure of the constructed vaccine, the PSIPRD web-based tool was used in this study (71). The PSIPRED web-based tool offers a user-friendly interface and employs a machine-learning approach to analyze protein sequences and predict their secondary structures. This tool also utilizes a cross-validation approach to validate its performance (72).

#### 2.6.2 Tertiary structure prediction and refinement

The three-dimensional (3D) structure of the final multiepitope vaccine construct was predicted by using the Iterative Threading ASSEmbly Refinement (I-TASSER) homology modeling server (45). The initial model of the MCV vaccine identified from the I-TASSER server was further validated and refined using the GalaxyRefine web server developed based on a refinement method that has been successfully implicated and investigated in CASP10 (46). Schrödinger Maestro (Schrödinger Release 2022-3: Maestro, Schrödinger, LLC) tools were used to visualize the obtained initial and refined 3D structure of the vaccine candidate.

#### 2.6.3 Structure validation

Validation of the protein structure that has been predicted through homology modeling is the core of structural determination methods. Validation of the protein 3D structure provides a more extraordinary idea about the compatibility of a structural model with its amino acid (AA) residues. It helps to determine the missing AA residues of the protein (73). Therefore, to validate the structural confirmation of the proposed MCV vaccine, the 3D structure of the protein was submitted to the ProSA-web server (47). The overall quality of the protein structure was accessed based on the z-score value provided by the server. If the z-scores of the anticipated model fall outside compared to the construction of the native protein, it indicates an erroneous protein. Additionally, the Ramachandran plot evaluation of the proposed vaccine candidate was performed by utilizing the Ramachandran Plot Server developed by ZLab to check the main-chain conformational tendencies of AA residues (74).

### 2.7 Molecular docking

Molecular docking is a very commonly used computational method that simulates the interaction of a ligand with its receptor and consequently forecasts the energy score generated during the interaction (75). The technique can determine the binding affinity of two molecules based on certain scoring functions. For molecular docking, the desired TLR4 receptor was retrieved from the RCSB Protein Data Bank (PDB) having a PDB ID: 4G8A. The TLR4 receptor was docked with the vaccine candidates that were defined as a ligand during the docking simulation. The TLR4 receptor was prepared by removing water and heteroatom and adding hydrogen through Schrödinger’s protein preparation wizard (76). To evaluate the binding affinity, molecular docking was performed by using ClusPro 2.0 web server (49). The performance of the server was assessed based on the ability to cluster the lowest energy structure, rigid body docking, and structural refinement process depending on energy minimization. The best-docked complex was selected and retrieved based on the binding affinity between the ligand–receptor complex. The interaction between the receptor TLR4 and vaccine construct was visualized by using the PyMOL visualization tool (77).

### 2.8 Complex structural stability evaluation through molecular dynamics simulation

The stability of the protein–protein complex refers to stable protein dynamics (more association and less dissociation of a protein–protein complex). The binding strength of the receptor and ligand (vaccine candidate) complex system and their dynamic behavior can be evaluated using different computational tools and animal model systems. To ascertain the constancy of the predicted vaccine and vaccine–receptor (VR) complex, a computational molecular dynamics (MD) simulation approach of the refined vaccine and VR complex was performed using ‘Desmond v6.3 Program’ in Schrödinger (Academic version) under the Linux operating system. The thermodynamic stability of the vaccine and VR complex was calculated using this computational approach, where a predefined TIP3P water model was used to emulate water molecules using the OPLS3e force field (78). Orthorhombic periodic boundary conditions were set up to specify the shape and size of the repetition unit safeguarded at 20 Å distances. To achieve electrical neutralization, the system was balanced by adding suitable sodium and chlorine ions, ensuring a minimized charge within the Desmond module. This process was carried out utilizing the OPLS3e force field. Molecular dynamic simulations were carried out with periodic boundary conditions in the constant number of particles, pressure, and temperature (NPT) ensemble (79). The temperature and pressure were kept at 300 K and 1 atm using Nose–Hoover temperature coupling and isotropic scaling (80). The operation was followed by running the 200 ns simulation and saving the configurations thus obtained at 200 ps intervals. The vaccine and vaccine complex stability was further evaluated using statistical parameters like root mean square deviation (RMSD), root mean square fluctuation (RMSF), the radius of gyration (rGyr), and hydrogen bond (HB) values. The superimposition of the vaccine and VR complexes was also evaluated in this study. The entire molecular dynamics (MD) simulation was executed in the Linux (Ubuntu-20.04.1 LTS) operating system and Intel Core i7-10700K processor CPU, 3200 MHz DDR4 RAM, and RTX 3080 DDR6 8704 CUDA core GPU.

### 2.9 Immune response simulation

*In silico* immune simulations were used to estimate the possible immunogenic profile of multiepitope vaccine candidates in real-life conditions by using the C-IMMSIM server (81). The output of the immune responses was salvaged for comprehensive observation. For ideal vaccine candidates, the minimum recommended interval between doses 1 and 2 is 3–4 weeks (22). Therefore, a minimum gap of 30 days between two dosages was taken into consideration in this study. Three injections of the vaccine candidates were administered computationally with time steps of 1, 84, and 168, where the one-time step was considered eight h in real life. The immune simulation was carried out for a total of 300 steps, and the rest of the simulation parameters were kept defaults.

### 2.10 Codon optimization and *in silico* cloning

Codon optimization is a gene engineering technique that employs synonymous codon modifications to enhance protein expression (82). Optimization of codon should be performed based on the specific host organism or expression system because the expression pattern of a foreign gene depends on the type of host organisms or expression system (53). To optimize the codon of the desired vaccine candidate, the JCat tool was used in this study (50). The tool uses an algorithm to maximize codons based on the codon adaptation index (CAI) (83). In this study, the widely used *E. coli* K12 was considered the host, and based on the expression system, codon optimization was performed. The following criteria were skipped during the optimization steps: (i) restriction enzyme (RE) cleavage sites, (ii) rho-independent termination of transcription, and (iii) binding sites of the prokaryotic ribosome. The final and optimized sequence was evaluated based on the CAI value and guanine–cytosine (GC) content. Finally, the optimized nucleotide sequence of the vaccine construct was inserted into the pET28a (+) vector using SnapGene 3.2.1 software.

## 3 Results of the study

### 3.1 Proteome retrieval and antigenicity prediction

The target sequence of MCV was retrieved in FASTA format from the UniProt database. Five proteins were recovered from the database: large T-antigen, small T-antigen, VP1, VP2, and VP3. The VaxiJen 2.0 and ANTIGENpro tools predicted the antigenic potency of the selected proteins listed in **Table 2**. All the primary sequences of the chosen protein have good antigenic properties that were used for further analysis.

**Table 2:** The selected proteins of MCV along with their corresponding antigenicity scores, which were identified using the VaxiJen 2.0 and ANTIGENpro tools.

NCBI ID	Protein Name	Antigenicity Score		Remark
		VaxiJen server	AnitgenPro server	
B6DVW7	Large T antigen	0.4762	0.889	Selected
B0G0V7	Small T antigen	0.5042	0.761	Selected
B0G0 W3	VP1	0.4374	0.942	Selected
B0G0 W4	VP 2	0.6649	0.697	Selected
A0A0N9DRI5	VP 3	0.5721	0.5	Selected

### 3.2 Epitope evaluation and selection

The selected five antigenic proteins with better antigenicity scores were submitted to a different server that predicted the different number of CTL, HTL, and linear BCL epitopes. Subsequently, the antigenic, immunogenic, toxic, and non-allergenic properties of the epitope’s candidates were evaluated, which found a high number of potential epitopes. However, we selected 30 (10 CTL, 10 HTL, and 10 linear BCL) epitopes for further evaluation. After considering the antigenic, immunogenic, and non-toxic properties, the selection process determined the best 10 epitopes for constructing a multiepitope vaccine against MCV. In the case of each antigenic protein found in MCV, two CTL epitopes, two HTL epitopes, and two linear BCL epitopes were explicitly chosen, listed in **Table 3**.

**Table 3:** The top two selected cytotoxic T lymphocyte (CTL), helper T lymphocyte (HTL), and linear BCL epitopes of MCV, as predicted by the NetCTL 1.2, IEDB MHC-II, and BepiPred 2.0 servers, respectively.

Protein name	CD8 epitope	CD4 epitope	Linear B-cell epitope
Large T antigen	LPFELGCAL	FKVDFKSRHACELGC	PEEPPSSRSSPR
	FELEFALDK	VIMMELNTLWSKFQQ	NKPLLNYEFQEK
Small T antigen	TLEETDYCL	CFCYQCFILWFGFPP	GCMLKQLRDSKC
	LNRKEREAL	VIMNELNTVFSKFQQ	CKLSRQHCSLKT
VP 1	PRYFNVTLR	CDTLQMWEAISVKTE	GLVLDYQTEYPK
	SVAPAAVTF	FNVTLRKRWVKNPYP	FAIGGEPLDLQG
VP 2	LVNYPASWV	AQLGFTAEQFSNFSL	GQDIFNSLSPTS
	QLGFTAEQF	ATTGVTLEAILTGKA	LAQLGFTAEQFS
VP 3	LVNRDVSWV	RHALMAFSLDPLQWE	NSRWVFQTTASQ
	QLGCLGEQF	VNLILNSRWVFQTTA	SLVNRDVSWVGS

#### 3.2.1 Potential cytotoxic T lymphocyte epitopes

Using the NetCTL v1.2 server, unique CTL epitopes (9-mer) were predicted from the MCV-selected five antigenic proteins. A total of 90 (29, 8, 16, 21, and 16 CTL epitopes from the large T-antigen, small T-antigen, VP1, VP2, and VP3, respectively) unique epitopes were identified that were antigenic, immunogenic, non-toxic, and non-allergenic (**Table S1**). The best two CTL epitopes for each protein (total 10) were selected and considered for further evaluation (**Table 3**).

#### 3.2.2 Potential helper T lymphocyte epitopes

A total of 47 unique HTL epitopes (15-mer) were predicted using the IEDB MHC-II prediction tool. Among the 47 unique epitopes, 6, 7, 13, 11, and 10 HTL epitopes were identified from the large T-antigen, small T-antigen, VP1, VP2, and VP3, respectively (**Table S2**). The epitopes were evaluated based on cytokine (IFN-γ, IL-4, and IL-10)-inducing ability and antigenic properties. Based on the aforementioned properties, a careful analysis was conducted, leading to the selection of the top two HTL epitopes for each protein. These epitopes were chosen for further evaluation and are presented in **Table 3**.

#### 3.2.3 Potential BCL epitopes

Specific antigenic regions of a protein that ultimately trigger antibody formation are known as BCL epitopes. The BepiPred 2.0 tool was used to predict linear B-cell (12-mer) epitopes from the selected proteins. A total of 70 (22, 8, 18, 12, and 10 epitopes from the large T-antigen, small T-antigen, VP1, VP2, and VP3, respectively) linear B-cell unique epitopes were identified, which were antigenic, non-allergenic, and non-toxic (**Table S3**). Here, we also selected the top two B-cell epitopes from each protein (total 10) for further evaluation (**Table 3**).

### 3.3 Worldwide population coverage

**Figure 2:**
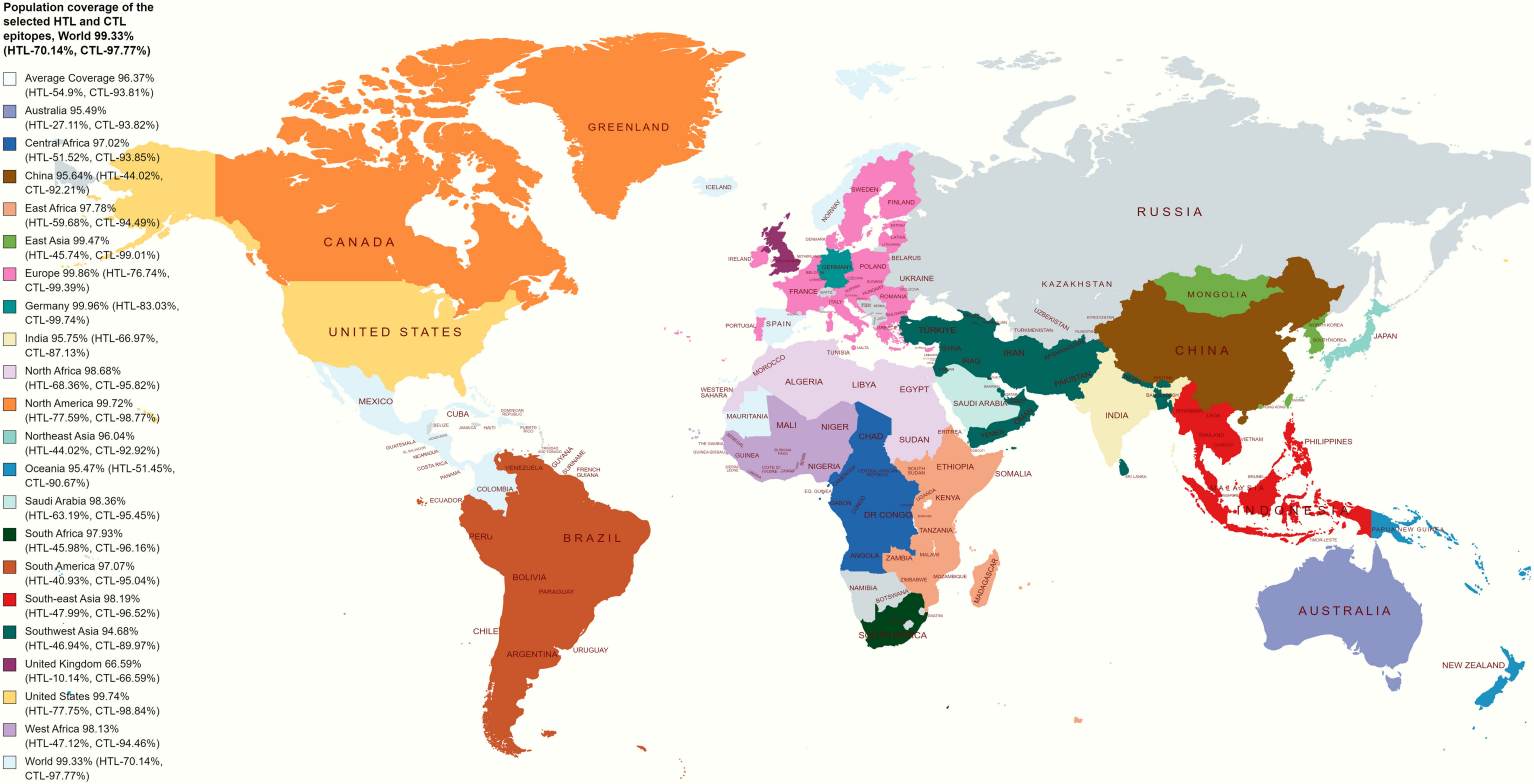
Illustrates a global population coverage map predicted using specific CTL and HTL epitopes. The map showcases the estimated coverage in various regions worldwide. These predictions rely on the chosen CTL and HTL epitopes, essential for stimulating cellular immune responses. The map offers valuable information about the potential effectiveness and coverage of the epitopes in diverse populations, assisting in evaluating and optimizing vaccine design strategies.

The worldwide population coverage ability of the vaccine candidates has been evaluated based on the selected CTL and HTL epitopes depicted in **Figure 2**. CTL and HTL epitopes showed a considerably high percentage (%) of population coverage. The combined world population coverage found for the CTL and HTL epitopes was 99.33%, where CTL individually shows a world coverage of 97.77% and HTL shows a world coverage of 70.14%. The identified epitopes are also prone to a high number of HLA alleles originating from different countries, such as Germany, Europe, the United States, South Asia, and India, with a combined (CTL and HTL) population coverage of 99.96%, 99.86%, 99.74%, 96.30%, and 95.75%, respectively (**Figure 2**). Therefore, the vaccine candidates that have been designed by utilizing the selected epitopes will cover most of the population around the world.

### 3.4 Formulation of multiepitope vaccine

**Figure 3:**
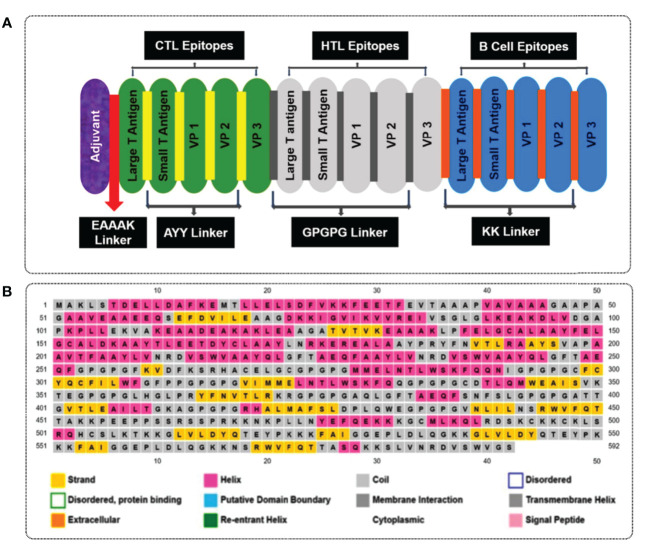
**(A)** A visual representation of the MCV vaccine constructs. Different colors are used to denote the adjuvant (purple), cytotoxic T lymphocyte (CTL, green), helper T lymphocyte (HTL, white), and linear B-cell epitope (linear BCL, blue) epitopes. The adjuvant and CTL epitopes are connected by the EAAAK linker (indicated in red), while AYY (gold color), GPGPG (gray boxes), and KK (orange boxes) linkers are employed to join the CTL, HTL, and linear BCL epitopes, respectively. **(B)** represents the secondary elements, including α-helices (pink), β-strands (yellow), and random coils (blue) of the MCV vaccine candidate.

To design multiple epitope vaccine candidates, initially, the 10 best highly antigenic CTL epitopes that were immunogenic, non-allergenic, and non-toxic were selected from each of the five (large T-antigen, small T-antigen, VP1, VP2, and VP3 of MCV) proteins (**Table 3**). Based on the cytokine-inducing properties, the best 10 HTL epitopes were selected from five proteins, which were highly antigenic and had the potential to generate cytokines. At last, the 10 best linear B-cell unique epitopes were identified from the structural protein of MCV, which were antigenic, non-allergenic, and non-toxic. The vaccine construct was formulated by using the selected 30 epitopes belonging to three different classes (10 CTL, 10 HTL, and 10 LBL). The vaccine constructs were initially accompanied by the TLR4 agonist 50S ribosomal protein L7/L12 as an adjuvant, positioned before the constructs connected to the first CTL epitope using EAAAK linkers. The selection of 30 epitopes, comprising 10 CTL, 10 HTL, and 10 BCL epitopes, was joined by the utilization of AAY, GPGPG, and KK linkers, respectively, to establish the desired connections between the epitopes. The total AA residue count in the final vaccine construct was 592. The sequential arrangement of the different epitopes and their corresponding linkers is shown in **Figure 3**.

### 3.5 Physicochemical and immunological properties of the vaccine

The ProtParam server was used to analyze the physicochemical properties of the multiepitope vaccine construct (**Table 4**). It exhibited an antigenic score of 0.6730, indicating a significant ability to elicit an immune response and effectively initiate interactions between antigens and antibodies. Based on the analysis, the vaccine candidate showed a molecular weight of 64,118.85 Da, which suggests a moderately sized construct. This size has implications for several important aspects of the vaccine’s development, including manufacturing, formulation, and stability (43). The theoretical isoelectric point that represents the pH of the vaccine was calculated to be 8.72, suggesting alkaline or basic nature of the construct. The alkaline nature of the construct has significant implications for various aspects such as its stability, solubility, and interactions with other molecules or components present in the formulation. The vaccine shows an instability index (II) of 30.77, which indicates a good post-expression stability of the construct. The thermostability of the construct was determined by assessing the aliphatic index, which yielded a value of 77.55. This range falls between 70 and 100, indicating that the proteins within the construct possess a notable degree of thermal stability. The server calculated the GRAVY as -0.210, which indicates a strong correlation with the highly hydrophilic nature of the construct. This hydrophilicity is expected to facilitate significant protein–protein interactions. The analysis also revealed that the vaccine had an estimated half-life of 30 h in mammalian reticulocytes in an *in vitro* setting. In yeast cells, the vaccine showed a half-life of over 20 h in an *in vivo* environment. Similarly, in *Escherichia coli*, the estimated half-life exceeded 10 h in an *in vivo* setting. These results suggest that the vaccine exhibits a comparatively long-lasting presence and stability across various biological systems, emphasizing its potential effectiveness and durability. Evaluation of immunogenicity provided a value of 1.24781. Moreover, the analysis of allergenicity properties shows the absence of allergenic features in the vaccine candidate. Additionally, the candidate showed a high solubility rate of 0.98246 as determined by the SOLpro server indicates that the candidate is expected to have good solubility in aqueous solutions (44). It implies that the vaccine construct has a high likelihood of dissolving well and remaining in solution, which is advantageous for its formulation and administration.

**Table 4:** List of the physiochemical parameters, antigenicity, immunogenicity, allergenicity, and solubility of the final vaccine candidates.

Parameters	Evaluation of properties
Number of amino acids	592
Molecular weight	64,118.85 Da
Theoretical pi	8.72
Total number of positively charged residues (Arg + Lys)	72
Total number of atoms	9,050
Extinction coefficient (at 280 nm in H2O)	82,570
Estimated half-life (mammalian reticulocytes, *in vitro*)	30 h
Estimated half-life (yeast cells, *in vivo*)	>20 h
Estimated half-life (*Escherichia coli*, *in vivo*)	>10 h
Instability index	30.77
Aliphatic index	77.55
Grand average of hydropathicity (GRAVY)	-0.210
Antigenicity	0.6730
Immunogenicity	1.24781
Allergenicity	Non-allergen
Solubility	0.982460

### 3.6 Vaccine structure prediction, refinement, and validation

#### 3.6.1 Secondary structure prediction

The secondary structures of the vaccine candidate were composed of extended strands, alpha helices, and random coils. The secondary structure of the vaccine construct was estimated *via* the PSIPRED 3.2 server. The analysis yielded an average Q3 score of 81.6% for the helix, sheet, and loop). The Q3 score serves as a valuable metric to assess the accuracy of secondary structure prediction methods like PSIPRED (71). It quantifies the proportion of correctly predicted secondary structure elements (helix, sheet, or loop) in relation to the known experimental structure of a protein. The study obtained a Q3 score of 81.6%, reflecting a high level of accuracy in predicting the secondary structure. This score signifies that approximately 81.6% of the amino acids (AA) in the construct were correctly assigned to their respective secondary structure elements (helix, sheet, or loop) by the prediction algorithm. Notably, our observations revealed a notable prevalence of alpha-helices in the construct, visualized by the pink color in **Figure 3B**. Alpha-helices are widely acknowledged for their remarkable structural stability and often play a critical role in protein folding and stability. Additionally, the presence of loops, depicted by the gray color, indicates flexible regions that contribute to conformational variability and can actively participate in protein–protein interactions and antigenic determinants. The construct consisted of 592 AAs in total, and the α-helix, β-strands, and random coils found in the structure indicated by pink, yellow, and gray colors, respectively, are represented in **Figure 3B**.

#### 3.6.2 Tertiary structure prediction

**Figure 4:**
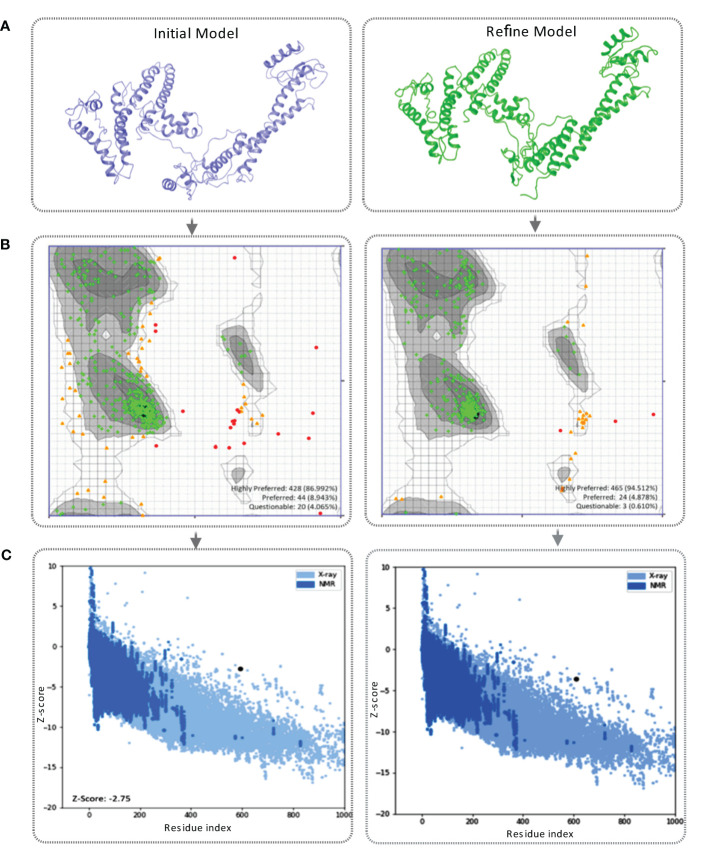
**(A)** This figure showcases the tertiary structure of the MCV vaccine model. The left side displays the initial model of the vaccine, while the right side represents the refined vaccine construct. **(B)** The Ramachandran plot of the final vaccine model, initial vaccine model (right), and refined vaccine model (left). Highly preferred conformations are represented by black, dark gray, and gray, while preferred conformations are depicted by white with a black grid. Questionable conformations are shown as white with a gray grid. **(C)** The validation of the final vaccine model was performed based on the Z-score. The Z-score for the initial model was -2.75, whereas the refined model attained a Z-score of -2.59.

The vaccine construct’s tertiary structure was generated using the I-TASSER server. The server provided the top five 3D models of the vaccine construct with different C-score values (**Table S4**). The C-score is a confidence score for estimating the quality of predicted models generated by I-TASSER. The study considered the model with the lowest C-score (–1.37), as recommended by the server and visualized by Schrodinger Maestro (**Figure 4A**).

#### 3.6.3 Tertiary structure refinement

The Galaxy Refine server was used to refine the projected tertiary structure of the final vaccine construct. The initial protein model retrieved from the I-TASSER was submitted for refinement. The protein-refining server provided five refined models with the presence of an increased number of AA residues in the favorable region listed in **Table S5**. The study selected the best refined model based on the Ramachandran favored score. In this study, model-5 (**Table S5**) shows a highly Ramachandran-favored score of 88.5% with a GDT-HA score of 0.9396, an RMSD value of 0.451, a MolProbity score of 2.363, and a clash score of 19 selected for further evaluation (**Table S5**). The refined vaccine model was visualized *via* Schrodinger Maestro represented in **Figure 4A**.

#### 3.6.4 Tertiary structure validation

The tertiary structure of the initial vaccine construct (before refinement) and final vaccine construct (after refinement) were validated by analyzing the output found from the Ramachandran Plot Server and ProSA-Web server. Ramachandran plot analysis of the initial vaccine model found that a total of 86.992% amino acid residues was in the favorable region of the plot (**Figure 4B**). However, after the refinement rampage server generated a Ramachandran plot, where a total of 94.512% of residues were in the favorable region of the plot (**Figure 4B**).

The prose-web server was used to assess the validation quality and potential errors in a crude tertiary structure model (**Table S6**). To validate the final vaccine model, its agreement with experimental data was assessed using the Z-score. The Z-score is a quantitative measure that evaluates the alignment between a model and experimental information. Its range varies depending on factors like the protein and its size. Generally, Z-scores fall within a range -4 to +4. When the Z-score approaches zero, it indicates a close resemblance of the model’s energy to experimentally determined structures (84). For the initial model, the Z-score was calculated as -2.75, indicating a moderate deviation from the experimental data. However, through refinement, the model achieved a slightly improved Z-score of -2.59 (**Figure 4C**). This suggests that the refined model exhibits better alignment with the experimental data, although the improvement is relatively minor.

### 3.7 Molecular docking

**Figure 5:**
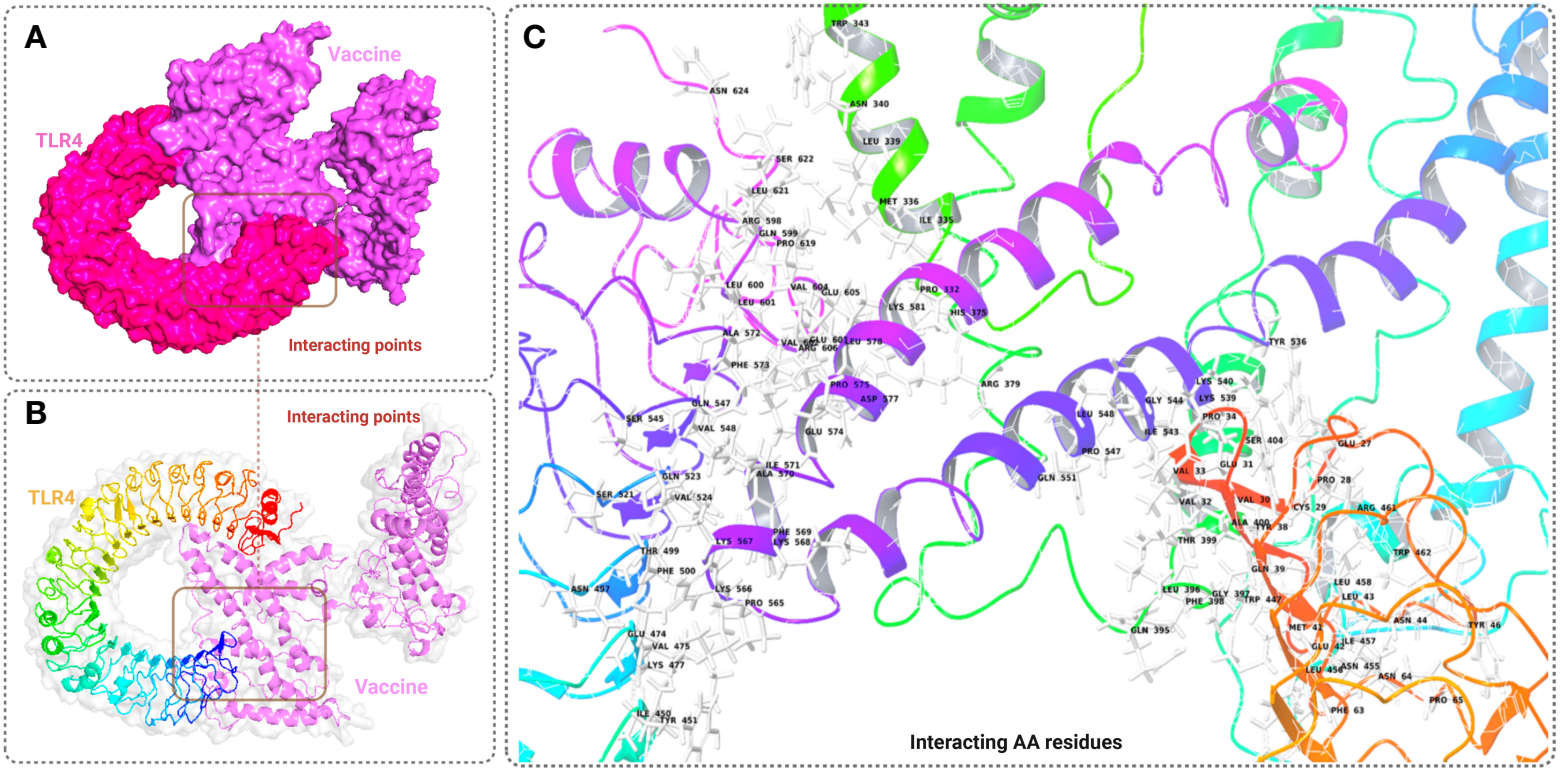
This figure depicts a graphical representation of the molecular interaction between the MCV vaccine candidates and the TLR-4 receptor. The molecular interaction is presented in three different views: **(A)** surface view, **(B)** cartoon view, and **(C)** specific amino acid interactions. The surface view in A provides an overall visual representation, while the cartoon view in B offers a simplified depiction. In C, specific amino acid interactions between the vaccine candidates and the TLR-4 receptor are highlighted.

The binding affinity of the receptor (TLR-4) and ligand (refined vaccine) was calculated by using the ClusPro 2.0 server. The server provided a total of nine complex confirmational structures along with different binding energy scores. The lowest and central energy of the cluster found for each complex structure is listed in **Table S7**. The best complex confirmation structure has been chosen based on the lowest energy value. In this study, Cluster-7 shows lowest binding energy value -1122.9 kcal/mol, which was retrieved for further analysis (**Figure 5A**). The interaction between the TLR-4 receptor and vaccine construct was analyzed from the VR docking complex and shown in **Figures 5B, C**. The interaction residue participant in the complex formation is also listed in **Table S8**.

### 3.8 Molecular dynamics simulation analysis

MD simulation is a convenient way that was used to analysis the structural stability of the vaccine and VR complex structure. The strength of the complex interface was evaluated based on RMSD, RMSF, Rg, intramolecular HBs (Intra HB), and ligand-protein contacts.

#### 3.8.1 Root mean square deviation of vaccine construct

**Figure 6:**
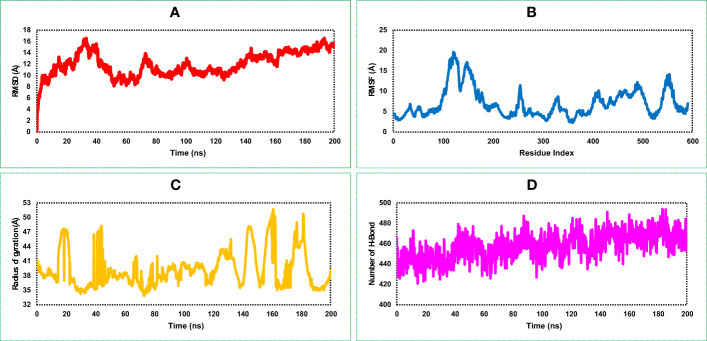
Representing four dynamic properties of the MCV vaccine construct obtained from a 200 ns molecular dynamics (MD) simulation. **(A)**, the root mean square deviation (RMSD) plot demonstrates the deviation of the vaccine construct from its initial conformation, indicating any structural changes or fluctuations during the simulation. **(B)** The root mean square fluctuation (RMSF) plot, revealing the residue-wise flexibility or fluctuation of the vaccine construct throughout the simulation. **(C)** The radius of gyration (Rg) quantifies the compactness or size of the vaccine construct during the simulation. **(D)** presents the number of HBs formed within the vaccine construct, highlighting the interactions and stability of the structure.

RMSD of the vaccine construct was measured to evaluate the average change happened due to the displacement of a selected atoms from the vaccine frame comparing to a reference frame. During the simulation of the vaccine construct, the highest fluctuation was 16.162 Å, the lowest was 3.109 Å, and the average was 11.94 Å (**Figure 6A**). A minor notch of fluctuation was observed for the vaccine structure after 160 ns dynamic simulation indicating structural stability of the vaccine construct.

#### 3.8.2 Root mean square fluctuation of vaccine construct

RMSF of the vaccine construct was calculated to examine the change of structural flexibility occurred due to the displacement of a specific AA residue in the protein. The RMSF plot of the vaccine construct showed a fluctuation peak between 95 and 570 AA residual positions. The highest fluctuation was 19.636 Å observed at GLU122 AA residual position, the second-highest fluctuation found at VAL124, and the third-highest fluctuation found at LYS123 AA residual position with an RMSF score of 19.413 and 18.95 Å, respectively, shown in **Figure 6B**. These fluctuations indicate regions of the protein that may have increased mobility or flexibility, potentially influencing its conformational changes, protein–protein interactions, and overall stability. These fluctuations are important for assessing the functional implications and optimizing the design and performance of the vaccine construct.

#### 3.8.3 Radius of gyration of vaccine construct

The distribution of atoms in the vaccine construct around its axis was measured based on the radius of gyration (Rg) value throughout the 200 ns simulation run. Analysis of the Rg profile found a higher deviation between 15 and 200 ns, where the average Rg score of the construct was 39.25 Å. The Rg score of the study provided information concerning the compactness of the vaccine. Herein, we found an average lower score of the Rg value vaccine construct indicating the tightest packing characteristic of α/β-proteins (**Figure 6C**).

#### 3.8.4 Number of hydrogen bonds of vaccine construct

Most of the direct contacts require protein folding, protein structure, and molecular recognition depends on the HBs of the structure. The number of HBs in a protein structure can be used to understand the protein structure and motions. Therefore, to understand the structure and motion of the vaccine candidate, the number of HBs found during the 200 ns simulations was analyzed and represented in **Figure 6D**. Analysis of the simulation data found the highest number of HBs between 90 and 200 ns simulation time. The average number of HBs found in this study was 457 for 200 ns simulation run indicating the vaccine construct will maintain active configurations by connecting protein structure in a fluxional equilibrium.

#### 3.8.5 Root mean square deviation of the vaccine–receptor complex structure

**Figure 7:**
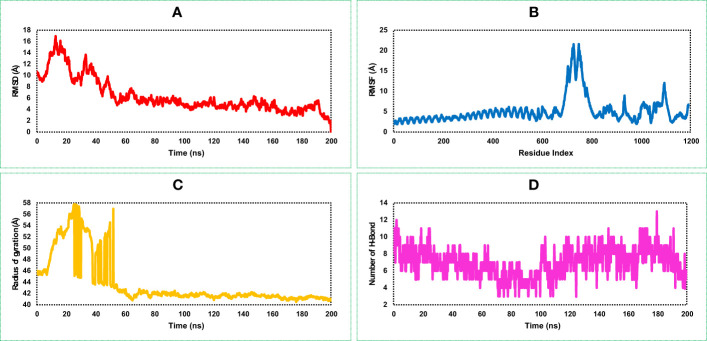
This figure illustrates the dynamic properties of the MCV vaccine–receptor (VR) (TLR-4) complex obtained from a 200 ns MD simulation. It examines the **(A)** RMSD plot, indicating structural changes and fluctuations in the MCV VR complex during the simulation. **(B)** displays the RMSF plot, revealing residue-wise flexibility and fluctuations within the complex. **(C)** quantifies the complex’s compactness and size using the radius of gyration. **(D)** highlights the interactions and stability of the structure through the number of hydrogen bonds (HBs).

The highest RMSD value of the VR complex found in this study was 16.918 Å. The VR complex structure shows the lowest and average RMSD value of 1.323 Å, and 6.34 Å, respectively during the 200 ns simulation run. The complex structure of the protein shows a stable and optimum fluctuation after 55 ns represented in **Figure 7A**. The RMSD of the vaccine complex structure (**Figure 7A**) was lower than the RMSD of the vaccine (**Figure 6A**) construct indicating stability of the complex structure.

#### 3.8.6 Root mean square fluctuation of the vaccine–receptor complex structure

Analysis of the RMSF plot of the VR complex found the most fluctuation peak between 650 and 800 AA residues represented in **Figure 7B**. The vaccine candidate that was in complex with the receptor showed the highest fluctuation between 700 and 710 AA residue with an average fluctuation of 20.1 Å. The second highest RMSF value was 8.57 Å found between 900 and 1,200 AA residual position, and the rest of the time, the VR complex shows an optimum fluctuation rate of a complex structure (**Figure 7B**). A comprehensive understanding and analysis of these RMSF fluctuations have played a critical role in the evaluation of the structural dynamics, ultimately facilitating the optimization of the MCV vaccine candidate’s design. This knowledge helped us to enhance the vaccine’s effectiveness and immunogenicity, leading to improved protective immune responses and potentially increasing its potential as a prophylactic measure against the MCV.

#### 3.8.7 Rg of vaccine–receptor complex structure

The VR-complex structure shows the average Rg value of 43.78 Å, and a high deviation of the score observed between the range of 5–55 ns simulation run. The lowest and stable Rg value of the VR complex was observed after 70–200 ns simulation run indicating tight packaging of the system (**Figure 7C**).

#### 3.8.4 Number of hydrogen bonds of the vaccine–receptor complex

The highest number of HBs found for the vaccine and receptor complex structure was between 0 and 20 ns and 120 and 200 ns simulation run (**Figure 7D**). The number of the HBs reduced in this study during 80–100 ns simulation run. However, throughout the 200 ns simulation, the complex structure of the protein consistently maintains an optimal number of hydrogen bonds (HB). This observation indicates the protein's significant contribution to the free energies of VR complexes.

### 3.9 Superimposition of vaccine and vaccine–receptor complex

**Figure 8:**
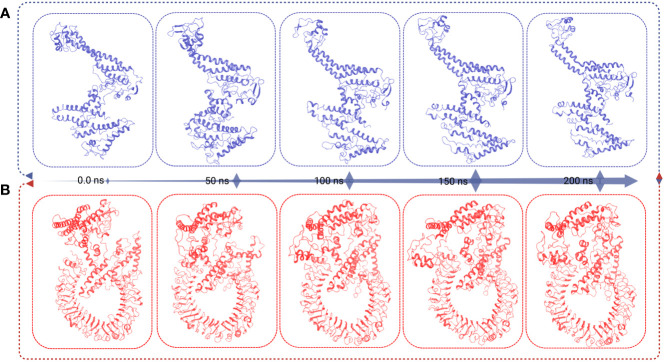
This figure presents the superimposition frames at different simulation times (0, 50, 100, 150, and 200 ns) for both the MCV vaccine and the vaccine receptor’s complex structure. **(A)** The superimposition frames illustrate the alignment and comparison of the MCV vaccine structure at different time points during the simulation. **(B)** showcases the superimposition frames of the complex structure formed between the MCV vaccine and its receptor, providing insights into their conformational changes and interactions over the simulation duration.

Different structural and conformational changes of the vaccine and VR complex were analyzed from the 200 ns simulation trajectory as shown in **Figure 8**. Conformational changes were observed for each 50 ns time interval during the simulation of the vaccine (**Figure 8A**) and the VR complex (**Figure 8B**). Very low confirmation change was found from the very beginning to 200 ns simulation time of the vaccine and VR complex. Therefore, the vaccine and vaccine complex remained stable in a 200 ns dynamic simulation trajectory (**Figure 8**).

### 3.10 Immune response analysis

**Figure 9:**
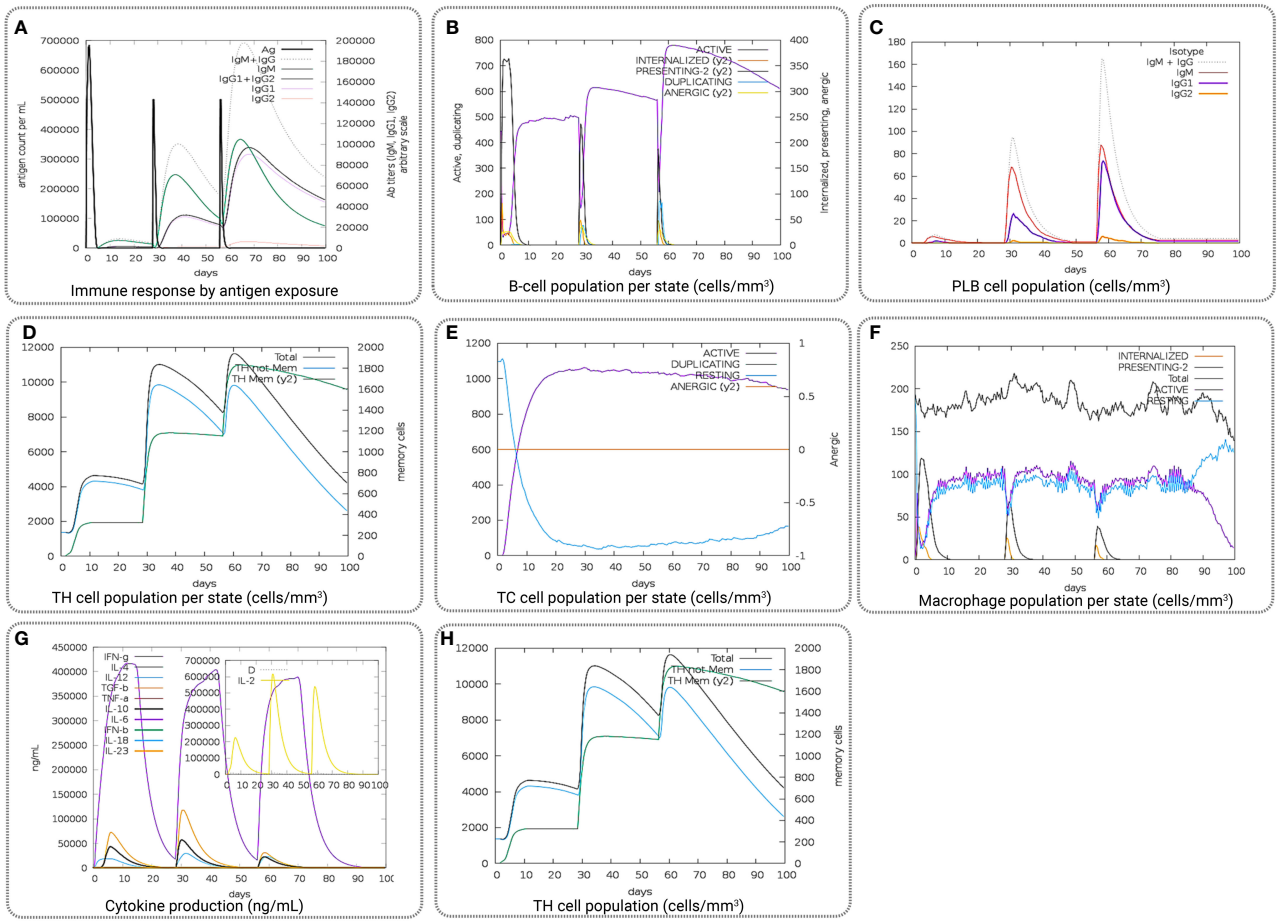
Representing the overall immune response of the vaccine candidate act as an antigen: **(A)** generation of immunoglobulins and B-cell isotypes upon exposure to an antigen; **(B)** amount of active B-cell populations per state; **(C)** amount of plasma B-lymphocytes and their isotypes per state; **(D)** state of helper T-cell population; **(E)** cytotoxic T-cell population per state of antigen exposure; **(F)** activity of macrophage population; **(G)** production of cytokine and interleukins in different states with the Simpson index, and **(H)** T.H. cell population (cells/mm^3^).

The computational immune simulation of the vaccine candidates found a response similar to the actual immune responses of a human, as shown in **Figure 9**. Secondary and tertiary responses generated during the immune simulation process were higher than the primary immune response. Analysis of the immune simulation initially identified higher concentrations of IgM in the case of the primary immune response, where the secondary and tertiary responses show higher levels of immunoglobulin activities (*i.e.*, IgG1 + IgG2, IgM, and IgG + IgM antibodies) with concomitant antigen reduction represented in **Figure 9A**. The results found in this study indicate the ability of the vaccine candidates to form memory T cells. The immune simulation also found some long-lasting B-cell isotypes that can help with potential isotype switching, resulting in the formation of memory cells (**Figures 9B, C**). In the case of TH (helper) and T.C. (cytotoxic) cells, a similar elevated response along with the respective memory development was also observed (**Figures 9D**, **E**). This indicates that the emergence of immune memory results in a high level of antigen clearance upon subsequent exposure (**Figure 9F**). During the exposure time, the immune system showed increased macrophage activity, with simultaneous proliferating dendritic cells (**Figures 9G, H**). High levels of IFN-γ and IL-2 were also observed during exposure, suggesting that it will help to promote the development of T regulatory cells. This profile suggests immune memory development and, therefore, natural immune protection against the virus (**Figure 9**).

### 3.11 Codon optimization and *in silico* cloning

**Figure 10:**
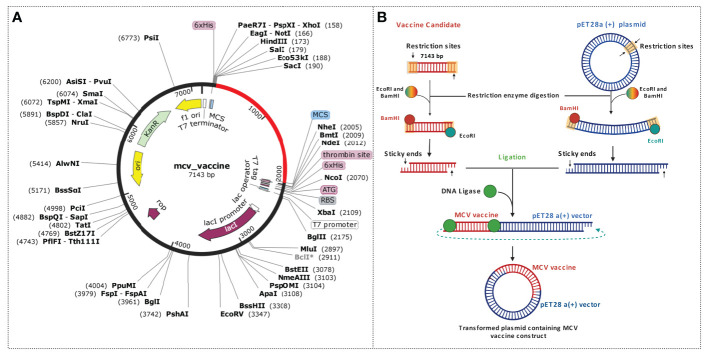
Schematic representation of the *in silico* cloning procedure for the MCV vaccine candidate into the pET28a (+) vector. **(A)** The coding gene sequence of the designed vaccine is depicted in red, while the vector backbone sequence of the designed vaccine is represented in black. **(B)** Illustration of the complete cloning process, encompassing restriction enzyme (RE) digestion and ligation steps.

This study utilized an *in silico* molecular cloning approach to analyze and modify the target vaccine sequence for compatibility with the selected vector. The process involved identifying suitable RE recognition sites, optimizing codon usage, and considering factors that could influence gene expression and protein production. Initially, the sequences of the vaccine candidate were optimized by using the codon optimization process to maximize the expression of the vaccine candidate in the *E. coli* K12 expression system. Codon optimization was performed by utilizing the 1,776 (bp) nucleotide sequences retrieved by converting the protein sequences of the construct. The task was completed by using the JCat tool and accessed based on the GC content and CAI value. The GC content found for the vaccine construct was 52. 36% lies between the normal range of 30%–70%. The CAI value found for the construct was 0.98, which also lies in the ideal range between 0.8 and 1.0. Based on the content of GC and CAI value, the MCV vaccine will be expressed highly whenever the *E. coli* expression system is utilized as a host. Two restriction digestion endonucleases, the EcoRI and BamHI, were used to cut the vaccine and vector pET28a (+) vector sequence (**Figure 10**). Herein, the cloned vaccine sequence’s absolute length was 7,143 bp after RE digestion and ligation, shown in **Figure 10A**. The steps and outcomes of this *in silico* molecular cloning process are illustrated in **Figure 10B**.

## 4 Discussion

Given the elevated mortality rate associated with MCC, exploring and advancing preventive measures have become an urgent and imperative matter (85). In such circumstances, vaccination is the most effective and suitable strategy for developing immunity against viral pathogens (32, 86). However, the conventional approach to designing and developing vaccines against viruses is financially demanding and time-consuming (87). It necessitates a complex and intricate selection process for identifying suitable immunodominant epitopes, antigens, and efficient delivery systems, presenting significant challenges and difficulties. With the advent of immunoinformatics and computational approach formulation of prophylactic vaccines against a specific disease or pathogens has become the fastest, easiest, and most cost-effective (70, 88). The immune system appears to play a critical role in MCC biology, with increasing evidence of virus-specific cellular and humoral immune responses that influence the prognosis of MCC patients (89). In recent decades, many treatment strategies have been applied to treat cancer, but the specific treatment option for the disease remains elusive (90). Previously, different peptide vaccines have served as promising anticancer candidates due to their ability to target tumor cells and induce specific T-cell responses (91, 92). Therefore, the study aimed to design a multiepitope peptide vaccine candidate to fight against MCC, a widely viral-causing skin cancer.

We predicted effective epitopes as antigens and their correspondence alleles for both B and T cells to generate a sufficient immune response against MCC-positive tumors (**Tables S9 and S10**). The study initially identified and retrieved the sequences of five MCV proteins: large T antigen, small T antigen, VP1, VP2, and VP3. These proteins play crucial roles in the MCV infection process and contribute to the understanding of the virus’ mechanisms (11). All five proteins (large T-antigen, small T-antigen, VP1, VP2, and VP3) exhibited notably high antigenicity scores. Consequently, we utilized all of these proteins to identify the most potent CD8, CD4, and linear B-cell epitopes, leading to the subsequent construction of a vaccine candidate targeting the virus. Several linear orders were applied to construct the multiepitope vaccine, and most potential vaccine structures were prepared by joining the adjuvant through the linker with CTL-HTL-BCL epitopes according to their higher to lower antigenic scores. The constructed vaccine candidate has a molecular weight of 64118.85 D with a theoretical PI of 8.72, indicating the basic properties of the protein. The aliphatic index provides insight into the relative presence of aliphatic side chains, such as alanine, valine, isoleucine, and leucine, within the protein structure. With a value of 77.55, the aliphatic index indicates a high level of thermal stability for the protein (87, 93). Additionally, the GRAVY value was determined to be -0.210, suggesting a hydrophilic nature of the construct and strong interactions with water molecules (88, 94). Finally, the study identified linear contiguous AA sequence fragments and confirmed AA fragments as potential epitopes for BCL that are immunogenic and antigen in nature and utilized for multiepitope vaccine design.

The 3D tertiary structure of the vaccine candidates was predicted and validated through different approaches. Subsequently, the vaccine candidates were refined, and structural validity was checked. The crude model of the vaccine candidates shows a Ramachandran score of 86.992% of the AA residues in the favorable region. After the refinement of the vaccine construct, the Ramachandran plot generated a better result of 94.512%, which means that most of the AA residues of the refined vaccine candidates were in the favorable regions. In addition, the Ramachandran plot shows that 94.512% of residues clustered tightly in the most favored region with very few residues in outliers. A good-quality model would probably exceed 90% in the most favored regions (95). The Z-score of the refined model was -2.59, indicating a satisfactory quality of the overall model. The Z-scores of the anticipated model were outside the scale of the property for local proteins, which shows the incorrect structure; thus, the MCV vaccine model is inside the scale property for local proteins (96, 97). Therefore, the structure of the vaccine candidate was deemed acceptable based on our evaluation. The length of the vaccine construct was determined to be 592 AA, acknowledging that the ideal length of a vaccine can vary based on factors including the target pathogen, desired immune response, and antigen or epitope characteristics (98). In the case of MCV, which exhibits genetic diversity and antigenic variability, it is often necessary to include multiple epitopes or larger antigenic regions to ensure comprehensive protection against various MCV strains (89). Incorporating multiple epitopes has the advantage of enhancing immune recognition, preventing immune evasion, and improving cross-reactivity and cross-protection (99). Therefore, the use of a 592 AA construct containing multiple epitopes supports an effective immune response and provides protection against MCV strains.

We also employed molecular docking simulation to determine the binding affinity between the vaccine candidate and the TLR-4 receptor (75, 100). We found that the vaccine can properly bind with the receptor TLR-4 and has the lowest binding energy score. A comprehensive structural analysis of the vaccine candidate and its receptor complex was also performed through MD simulation approaches to determine the binding stability of the complex system (76, 101). The vaccine conformation showed an average RMSD change of 11.94 Å, with fluctuations ranging from 3.109 Å to 16.162 Å. RMSF analysis identified peak fluctuations between amino acid residues 95–570, mainly at GLU122, VAL124, and LYS123. The compactness of the structure was confirmed by radius of gyration (Rg) analysis, which yielded an average Rg score of 39.25 Å. Analysis of HBs revealed a significant increase, peaking between 90 and 200 ns simulation time. For the vaccine–receptor complex, stable fluctuations were observed after 55 ns with an RMSD value of 16.918 Å. RMSF analysis highlights fluctuations between amino acid residues 650–800, particularly at position 700–710. The Rg analysis shows tight packing with deviation observed from the 5–55 ns simulation run (102). The number of hydrogen bonds was observed to fluctuate, with the highest number observed between 0 and 20 ns and 120 and 200 ns simulation time. Simulated microscale changes in the protein backbone and mild fluctuations of the side chain residues were observed in this study, which altogether confirmed the stability of the vaccine–TLR4 complex. These findings deepen our understanding of the conformation of the vaccine and the structural dynamics and interactions within its receptor complex. Stable and fluctuating regions provide valuable insights into conformational changes and intermolecular associations, facilitating further optimization and development of vaccines.

In addition, the immunological response of the vaccine candidate was also evaluated, which showed higher B- and T-cell activity, indicating the typical immune response. The evaluation of the immune response was performed by using the vaccine as an antigen, where a high level of immunoglobulin and B-cell isotype formation was observed upon exposure. Upon exposure, the number of active B-cell populations, plasma B-lymphocytes and their isotype, helper T-cell and cytotoxic T-cell population per state; the number of plasma B-lymphocytes and their isotypes per state; state of helper T-cell population; and cytotoxic T-cell population, macrophage population, production of cytokine and interleukins per state were improved substantially, indicating a better memory formation ability of the vaccine candidates. In the final stage, the vaccine candidates underwent computational cloning into the pET28a (+) plasmid vector. Subsequently, the recombinant vaccine constructs were subjected to *in silico* expression within the *E. coli* K12 expression systems for subsequent analysis and evaluation. Before being computationally expressed into the host system, the vaccine candidate was optimized through the codon adaptation method (83). The GC content of the sequence was 52.36% in the optimized DNA sequence, indicating the optimal range (30%–70%) for expression (103). Additionally, the CAI value of the sequence was 0.98, close to 1.0, which indicates the higher expression probability of the vaccine candidate in the expression vector (104). Consequently, adequate adaptation was accomplished for the large-scale production of vaccine candidates.

Previously, programmed death-1 (PD-1) cell surface receptor inhibitors have found as a valuable treatment option for MCC, particularly in cases where cancer has spread or is not responsive to other therapies. Although Programmed death ligand 1 (PD-L1) blockade is highly effective, ~50% of infected patients with skin carcinoma either do not respond to PD-L1 therapy or develop PD-L1 refractory disease and, thus, do not experience long-term benefit (105, 106). Few other studies performed *in silico* analysis and constructed multiepitope peptide vaccines, although it is known that Merkel cell polyoma–mediated skin cancer is caused by the pathogenic proteins including the large T antigen, small T antigen, and viral capsid proteins (V.P. 1, V.P. 2, and Vp3) and all are involved in viral pathogenicity (107). However, the previous study has only either targeted only VP1 or T-antigenic proteins, for the epitope selection (31, 32). The current study identified and selected epitopes from all major pathogenic and antigenic proteins that will increase the efficacy of the vaccine candidates. Eventually, the study formulated a multiepitope vaccine candidate that will help to fight against MCV and boost the immune system of humans.

## 5 Conclusion

Recent groundbreaking developments in immunoinformatics have introduced novel techniques for disease prevention. Considering past outbreaks of viral infections in humans, computational approaches have been embraced to identify swift treatment strategies for diverse viral diseases. Peptide vaccines currently expressed as the most successful treatment option for viral infections can be designed by using either free peptides or peptides coated on dendritic cells. At this instant, peptide-based vaccines that are being designed by computational methods can play a critical role in the treatment of different infectious viral diseases. MCC is an aggressive infectious disease for which effective vaccine candidates are not available, and hence, it is necessary to develop an effective vaccine candidate. Therefore, in this study, we designed and identify a potential peptide vaccine candidate against MCV by using computational approaches that can be further utilized for subsequent vaccine construction. The study successfully identified peptide candidates against the virus and designed a valid multiepitope vaccine construct to fight against the aggressive MCC caused by MCV. However, further *in vitro* and *in vivo* investigations are suggested to finally determine to ensure the candidate vaccine’s true potential in combating against MCV.

## Data availability statement

The original contributions presented in the study are included in the article/**Supplementary Material**. Further inquiries can be directed to the corresponding authors.

## Author contributions

FM, AA, and FA designed the project; RI, AS, MA, and RA performed the analysis of data; RI, AS, FM, RA, MA, and FA evaluated and interpreted the results; RI, RA, MA, and AS prepared the draft manuscript; RI, AS, RA, and FA performed data curation and visualization; FM, RI, AA, MA, and FA. critically reviewed and finalized the manuscript; FM, FA, and AA performed investigation and supervision of the manuscript. All authors contributed to the article and approved the submitted version.
