# Supplementary material for: Computational formulation of a multiepitope vaccine unveils an exceptional prophylactic candidate against Merkel cell polyomavirus
Source: Front Immunol. 2023 Jun 27;14:1160260. doi: 10.3389/fimmu.2023.1160260 (PMC10333698; doi:10.3389/fimmu.2023.1160260)
Supplement: Supplementary file 1 [file DataSheet_1.pdf]

Supplementary File

Article

Computational formulation of a multiepitope vaccine unveils an exceptional prophylactic candidate against Merkel Cell Polyomavirus

Authors: Raihan Rahman Imon [1,2], Abdus Samad [1,2], Rahat Alam[1,2], Ahad Amer Alsaiari [3], Md. Enamul Kabir Talukder[1, 2], Mazen Almehmadi [3], Foysal Ahammad [1, 4,*] Farhan Mohammad [5,*]

Table S1. List of CTL epitopes identified through the NetCTL 1.2 server. This table provides a compilation of CTL (Cytotoxic T Lymphocyte) epitopes that have been identified using the NetCTL 1.2 server. The epitopes listed in the table are potential targets for the immune system's cytotoxic T cells, which play a crucial role in recognizing and eliminating infected or abnormal cells.

Protein Name	Super type	CD8 Epitopes	Combined score	Antigenicity	Immunogenicity	Toxicity	Allergenicity
Large T Antigen	B7	LPFELGCAL	1.6136	1.8459	0.13721	Non-Toxin	No
	B44	FELGCALDK	0.6586	1.3245	0.01843	Non-Toxin	No
	B8	MLEMTRTEM	0.7323	1.2524	0.03358	Non-Toxin	No
	A26	DFPIDLSDY	0.853	1.0524	0.01482	Non-Toxin	No
	A3	ALIDLLEGK	0.685	1.0417	0.14858	Non-Toxin	No
	B62	IILAHYLDF	1.0038	1.0399	0.06739	Non-Toxin	No
	B8	MEIRKRRIL	1.3538	0.9923	0.05544	Non-Toxin	No
	B39	QKCENRSRL	0.6838	0.9815	0.01629	Non-Toxin	No
	A2	ELGCILLFI	0.7231	0.9467	0.13495	Non-Toxin	No
	B58	KAIELYDKI	0.7146	0.9209	0.02239	Non-Toxin	No
	A2	ILAHYLDFA	1.0399	0.889	0.11833	Non-Toxin	No
	B58	CIVTANDYF	0.5843	0.8763	0.10103	Non-Toxin	No
	B8	IPKYRNIWF	1.6925	0.8344	0.21233	Non-Toxin	No
	A1	RTDGTWEDL	0.9739	0.8291	0.38507	Non-Toxin	No
	A24	SFAAALIDL	0.5895	0.8285	0.20501	Non-Toxin	No
	A2	TLIARFSYT	0.6806	0.8267	0.10139	Non-Toxin	No
	A26	YYMGGVAWY	1.3114	0.8149	0.2114	Non-Toxin	No
	B27	GKTSFAAAL	0.5996	0.7911	0.05284	Non-Toxin	No
	A1	KTVSCFAIY	1.399	0.7887	0.01541	Non-Toxin	No
	A2	LLLCLIWCL	1.3723	0.7823	0.21207	Non-Toxin	No
	A24	AYEYGPNPY	0.7124	0.7207	0.0394	Non-Toxin	No
	A3	ILLFITLSK	1.7551	0.6829	0.17432	Non-Toxin	No
	B58	KTSFAAALI	0.9505	0.6439	0.16557	Non-Toxin	No
	B27	LQSEISYGK	0.9619	0.6202	0.0376	Non-Toxin	No
	A24	EYELDDHFI	1.1223	0.5987	0.15952	Non-Toxin	No
	B44	YELDDHFII	1.7253	0.5945	0.24733	Non-Toxin	No
	B7	TPVPTDFPI	1.0486	0.5657	0.15324	Non-Toxin	No
	B58	NSSRTDGTW	1.6579	0.5497	0.10834	Non-Toxin	No
	A24	TFCTISFLI	1.7694	0.5319	0.08775	Non-Toxin	No
Small T Antigen	A2	TLEETDYCL	0.8193	1.6017	0.15731	Non-Toxin	No
	B8	LNRKEREAL	1.3115	1.3174	0.05338	Non-Toxin	No
	B62	MQSGYNARF	1.4144	1.2188	0.03397	Non-Toxin	No
	A1	LLEIAPNCY	1.929	1.2106	0.15712	Non-Toxin	No
	B7	FPWEEYGTL	1.3913	0.9925	0.31795	Non-Toxin	No
	A26	ESFDWWQKT	0.6996	0.9833	0.26077	Non-Toxin	No
	A1	QKTLEETDY	0.5521	0.9298	0.23891	Non-Toxin	No
	A24	GFPPTWESF	1.2926	0.8231	0.21939	Non-Toxin	No
VP 1	B27	PRYFNVTLR	0.8288	1.3707	0.17544	Non-Toxin	No
	B58	SVAPAAVTF	1.5105	1.1863	0.13399	Non-Toxin	No

	A2	RVHDYGAGI	0.7312	1.1497	0.11394	Non-Toxin	No
	B7	APAAVTFQS	0.7152	1.0251	0.15993	Non-Toxin	No
	B58	LINVHYWDM	0.5928	0.8356	0.26736	Non-Toxin	No
	A3	RYFNVTLRK	1.4938	0.8273	0.12911	Non-Toxin	No
	B7	NPYPVVNLI	0.7378	0.7896	0.05476	Non-Toxin	No
	A2	SITQIELYL	0.9549	0.7854	0.10837	Non-Toxin	No
	B62	MFAIGGEPL	0.7264	0.7418	0.28954	Non-Toxin	No
	B58	ISCADIVGF	1.062	0.7271	0.22339	Non-Toxin	No
	A3	TIETVLGRK	0.9561	0.6477	0.16016	Non-Toxin	No
	A1	DLQGLVLDY	1.0655	0.6163	0.02816	Non-Toxin	No
	B39	AYSVARVSL	1.2699	0.6092	0.01284	Non-Toxin	No
	B44	HDYGAGIPV	0.584	0.5494	0.20874	Non-Toxin	No
	A26	DIVGFLFKT	0.6542	0.5452	0.12386	Non-Toxin	No
	A24	QFSNTLTTV	0.6113	0.5274	0.02259	Non-Toxin	No
**VP 2**	A2	LVNRDVSWV	0.5787	1.7746	0.10024	Non-Toxin	No
	B62	QLGFTAEQF	0.7469	1.7227	0.22025	Non-Toxin	No
	B58	SLVNRDVSW	0.9861	1.6956	0.01635	Non-Toxin	No
	A2	ALAQLGFTA	0.8912	1.531	0.03872	Non-Toxin	No
	B58	LILNSRWVF	1.0199	1.3377	0.08857	Non-Toxin	No
	B58	VNLILNSRW	0.5437	1.1966	0.00405	Non-Toxin	No
	A3	ILNSRWVFQ	0.8492	1.111	0.19358	Non-Toxin	No
	A2	GISGIEALA	0.5023	0.9731	0.23079	Non-Toxin	No
	B8	PLQWENSLL	0.5057	0.9663	0.1306	Non-Toxin	No
	B62	QQQTPDWLL	0.7875	0.8904	0.192	Non-Toxin	No
	A3	LVNLILNSR	0.807	0.8785	0.00378	Non-Toxin	No
	A2	ALAALEAEI	0.9763	0.832	0.22704	Non-Toxin	No
	A2	NIGEIATEL	0.7031	0.8318	0.36944	Non-Toxin	No
	A26	EGISGIEAL	1.086	0.8148	0.14237	Non-Toxin	No
	B44	IEGISGIEA	0.5005	0.7872	0.18654	Non-Toxin	No
	B62	VNQGLTYGF	0.7514	0.7409	0.03892	Non-Toxin	No
	A2	ALEAEISSL	1.0065	0.7407	0.05837	Non-Toxin	No
	A26	EIATELSAT	0.7845	0.5856	0.02206	Non-Toxin	No
	A26	QTPDWLLPL	1.4767	0.5782	0.20814	Non-Toxin	No
	B7	DPLQWENSL	0.6463	0.5292	0.08767	Non-Toxin	No
	A24	TYGFILQTV	1.3978	0.5058	0.17288	Non-Toxin	No
**VP 3**	A2	LVNRDVSWV	0.5787	1.7746	0.10024	Non-Toxin	No
	B62	QLGFTAEQF	0.7469	1.7227	0.22025	Non-Toxin	No
	B58	SLVNRDVSW	0.9861	1.6956	0.01635	Non-Toxin	No
	A2	ALAQLGFTA	0.8912	1.531	0.03872	Non-Toxin	No
	B62	LILNSRWVF	0.8835	1.3377	0.08857	Non-Toxin	No
	B58	VNLILNSRW	0.5437	1.1966	0.00405	Non-Toxin	No
	A3	ILNSRWVFQ	0.8491	1.111	0.19358	Non-Toxin	No
	A2	GISGIEALA	0.5023	0.9731	0.23079	Non-Toxin	No
	A24	QQQTPDWLL	0.7521	0.8904	0.192	Non-Toxin	No
	A3	LVNLILNSR	0.7975	0.8785	0.00378	Non-Toxin	No
	A26	EGISGIEAL	1.086	0.8148	0.14237	Non-Toxin	No
	B44	IEGISGIEA	0.5005	0.7872	0.18654	Non-Toxin	No
	B62	VNQGLTYGF	0.7514	0.7409	0.03892	Non-Toxin	No
	A26	QTPDWLLPL	1.4767	0.5782	0.20814	Non-Toxin	No
	B7	DPLQWENSL	0.6457	0.5292	0.08767	Non-Toxin	No
	A24	TYGFILQTV	0.8316	0.5058	0.17288	Non-Toxin	No

Table S2. List of HTL epitopes predicted through IEDB MHC-II server. This table presents a comprehensive list of HTL (Helper T Lymphocyte) epitopes that have been predicted using the IEDB MHC-II server. HTL epitopes play a crucial role in activating and coordinating immune responses by interacting with MHC class II molecules on antigen-presenting cells and assisting in the activation of B cells and cytotoxic T cells.

Protein Name	Epitopes (Start - End Position)	Common Core	Interferon -γ	Interleukin -4	Interleukin -10	Antigenicity
**Large T Antigen**	355-369	FKVDFKSRHACELGC	POSITIVE	IL4 inducer	IL10 inducer	2.0369
	351-365	KIEKFKVDFKSRHAC	POSITIVE	IL4 inducer	IL10 inducer	1.0716
	350-364	DKIEKFKVDFKSRHA	POSITIVE	IL4 inducer	IL10 inducer	0.6261
	787-801	EAGQDPLLNILIEEE	POSITIVE	IL4 inducer	IL10 inducer	0.1581
	52-66	MMELNTLWSKFQQNI	POSITIVE	IL4 inducer	IL10 inducer	0.3398
	50-64	VIMMELNTLWSKFQQ	POSITIVE	IL4 inducer	IL10 inducer	0.0836
Small T Antigen	147-161	CFCYQCFILWFGFPP	POSITIVE	IL4 inducer	IL10 inducer	0.7723
	146-160	ECFCYQCFILWFGFP	POSITIVE	IL4 inducer	IL10 inducer	0.5824
	145-159	GECFCYQCFILWFGF	POSITIVE	IL4 inducer	IL10 inducer	0.583
	144-158	LTWGECFCYQCFILW	POSITIVE	IL4 inducer	IL10 inducer	0.3269
	143-157	TWGECFCYQCFILWF	POSITIVE	IL4 inducer	IL10 inducer	-0.0638
	142-156	VIMMELNTLWSKFQQ	POSITIVE	IL4 inducer	IL10 inducer	0.2348
	50-64	WGECFCYQCFILWFG	POSITIVE	IL4 inducer	IL10 inducer	-0.0359
VP1	110-124	CDTLQMWEAISVKTE	POSITIVE	IL4 inducer	IL10 inducer	0.5533
	111-125	FNVTLRKRWVKNPYP	POSITIVE	IL4 inducer	IL10 inducer	0.5322
	112-126	LHGLPRYFNVTLRKR	POSITIVE	IL4 inducer	IL10 inducer	0.7487
	306-320	HGLPRYFNVTLRKRW	POSITIVE	IL4 inducer	IL10 inducer	0.5617
	305-319	VTLRKRWVKNPYPVV	POSITIVE	IL4 inducer	IL10 inducer	0.5461
	304-318	NVTLRKRWVKNPYPV	POSITIVE	IL4 inducer	IL10 inducer	0.5013
	303-317	TCDTLQMWEAISVKT	POSITIVE	IL4 inducer	IL10 inducer	0.4383
	302-316	DTLQMWEAISVKTEV	POSITIVE	IL4 inducer	IL10 inducer	0.3835
	301-316	PRYFNVTLRKRWVKN	POSITIVE	IL4 inducer	IL10 inducer	0.3649
	300-315	YFNVTLRKRWVKNPY	POSITIVE	IL4 inducer	IL10 inducer	0.3539
	299-314	RYFNVTLRKRWVKNP	POSITIVE	IL4 inducer	IL10 inducer	0.2861
	297-313	LPRYFNVTLRKRWVK	POSITIVE	IL4 inducer	IL10 inducer	0.2199
	296-310	TLRKRWVKNPYPVVN	POSITIVE	IL4 inducer	IL10 inducer	0.1327
VP2		AQLGFTAEQFSNFSL	POSITIVE	IL4 inducer	IL10 non-inducer	1.6288
		TAEQFSNFSLVASLV	POSITIVE	IL4 inducer	IL10 non-inducer	0.9783
		ATTGVTLEAILTGKA	NEGATIVE	IL4 inducer	IL10 inducer	0.7807
		TTGVTLEAILTGKAL	NEGATIVE	IL4 inducer	IL10 inducer	0.5259

		TGKALAALEAEISSL	NEGATIVE	IL4 inducer	IL10 inducer	0.6672
		LVNQGLTYGFILQTV	POSITIVE	Non IL4 inducer	IL10 inducer	0.5917
		SLVNQGLTYGFILQT	POSITIVE	Non IL4 inducer	IL10 inducer	0.5953
		KALAALEAEISSLMT	NEGATIVE	IL4 inducer	IL10 inducer	0.8729
		LEAEISSLMTIEGIS	NEGATIVE	IL4 inducer	IL10 inducer	0.6966
		TLLANIGEIATELSA	POSITIVE	Non IL4 inducer	IL10 inducer	0.4839
		VNQGLTYGFILQTVS	POSITIVE	Non IL4 inducer	IL10 inducer	0.8744
VP3		RHALMAFSLDPLQWE	Positive	IL4 inducer	IL10 inducer	1.1319
		HALMAFSLDPLQWEN	Positive	IL4 inducer	IL10 inducer	1.0673
		VNLILNSRWVFQTTA	Positive	IL4 inducer	IL10 inducer	1.0053
		ALMAFSLDPLQWENS	Positive	IL4 inducer	IL10 inducer	0.9784
		LNSRWVFQTTASQNQ	Positive	IL4 inducer	IL10 inducer	0.8909
		LILNSRWVFQTTASQ	Positive	IL4 inducer	IL10 inducer	0.8604
		NLILNSRWVFQTTAS	Positive	IL4 inducer	IL10 inducer	0.8582
		ILNSRWVFQTTASQN	Positive	IL4 inducer	IL10 inducer	0.8138
		NSRWVFQTTASQNQG	Positive	IL4 inducer	IL10 inducer	0.6875
		GQNIFNSLSPTSRLQ	Positive	IL4 inducer	IL10 inducer	0.525

Table S3. List of linear B cell epitopes predicted through the BCpred 2.0 server. This table presents a comprehensive list of linear B cell epitopes that have been predicted using the BCpred 2.0 server. Linear B cell epitopes are specific sequences of amino acids within a protein that can be recognized by antibodies produced by B cells. The BCpred 2.0 server employs computational algorithms to predict potential linear B cell epitopes based on their antigenicity and propensity to form accessible surface regions.

Protein Name	Start Position	Epitope	Probability Score	Antigenicity	Allergenicity	Toxicity
**Large T Antigen**	230	PEEPPSSRSSPR	0.88	0.05	No	Non-Toxin
	423	NKPLLNYEFQEK	0.83	0.9099	No	Non-Toxin
	144	GYGSFSASQASD	0.83	0.7407	No	Non-Toxin
	570	KKLQKIIQLLTE	0.81	0.9073	No	Non-Toxin
	600	TSFAAALIDLLE	0.79	0.5016	No	Non-Toxin
	72	DFSMFDEVDEAP	0.78	1.0664	No	Non-Toxin
	582	NIPKYRNIWFKG	0.78	0.6887	No	Non-Toxin
	351	KIEKFKVDFKSR	0.76	0.7404	No	Non-Toxin
	102	GKAYEYGPNPYG	0.73	0.7245	No	Non-Toxin

	170	EPTSSSGSSSRE	0.72	0.7086	No	Non-Toxin
	135	PPHSQSSSSGYG	0.71	0.6098	No	Non-Toxin
	4	VLNRKEREALCK	0.7	2.1592	No	Non-Toxin
	94	WRSGGFSFGKAY	0.68	0.5604	No	Non-Toxin
	592	KGPINSGKTSFA	0.68	0.7426	No	Non-Toxin
	293	SASFTSTPPKPK	0.66	0.5318	No	Non-Toxin
	106	EYGPNPYGTNSR	0.66	0.8716	No	Non-Toxin
	578	LLTENIPKYRNI	0.65	0.7997	No	Non-Toxin
	181	EETTNSGRESST	0.65	0.6778	No	Non-Toxin
	418	KLLQENKPLLNY	0.64	0.7313	No	Non-Toxin
	115	NSRSRKPSSNAS	0.61	0.5791	No	Non-Toxin
	530	EMLCKKFKKHLE	0.59	0.7418	No	Non-Toxin
	605	ALIDLLEGKALN	0.51	0.8115	No	Non-Toxin
**Small T Antigen**	108	GCMLKQLRDSKC	0.82	0.2614	No	Non-Toxin
	124	CKLSRQHCSLKT	0.76	0.2812	No	Non-Toxin
	4	VLNRKEREALCK	0.7	0.6533	No	Non-Toxin
	78	EVSTKFPWEEYG	0.62	0.84	No	Non-Toxin
	72	DFSMFDEVSTKF	0.62	0.9923	No	Non-Toxin
	63	QQNNHKLRSDFS	0.61	0.279	No	Non-Toxin
	113	QLRDSKCACISC	0.57	1.2848	No	Non-Toxin
	82	KFPWEEYGTLKD	0.54	0.459	No	Non-Toxin
**VP 1**	171	GLVLDYQTEYPK	0.8	0.4148	No	Non-Toxin
	222	IEVWCPDPSKNE	0.79	0.4341	No	Non-Toxin
	160	FAIGGEPLDLQG	0.79	1.0151	No	Non-Toxin
	166	PLDLQGLVLDYQ	0.77	1.075	No	Non-Toxin
	71	YTYTYDLQPKGS	0.75	0.7849	No	Non-Toxin
	197	RKMTPKNQGLDP	0.74	1.2098	No	Non-Toxin
	238	YGSIQTGSQTPT	0.72	0.5233	No	Non-Toxin
	276	LFISCADIVGFL	0.71	0.0555	No	Non-Toxin
	66	TTSNWYTYTYDL	0.7	0.2696	No	Non-Toxin
	244	GSQTPTVLQFSN	0.7	0.6787	No	Non-Toxin
	290	TSGKMALHGLPR	0.69	0.2842	No	Non-Toxin

	152	VSGVNYHMFAIG	0.68	0.9954	No	Non-Toxin
	302	YFNVTLRKRWVK	0.6	0.5353	No	Non-Toxin
	55	PRMGVNSPDLPT	0.59	0.1091	No	Non-Toxin
	204	QGLDPQAKAKLD	0.58	1.2182	No	Non-Toxin
	115	QMWEAISVKTEV	0.56	0.583	No	Non-Toxin
	95	YSVARVSLPMLN	0.52	0.3669	No	Non-Toxin
	90	ENLPAYSVARVS	0.52	0.2779	No	Non-Toxin
**VP 2**	147	GQDIFNSLSPTS	0.81	0.4983	No	Non-Toxin
	57	LAQLGFTAEQFS	0.78	1.3808	No	Non-Toxin
	14	IATELSATTGVT	0.72	0.8139	No	Non-Toxin
	101	VRLSREQVSLVN	0.72	0.661	No	Non-Toxin
	130	AFSLDPLQWENS	0.69	1.1795	No	Non-Toxin
	45	LMTIEGISGIEA	0.67	0.7853	No	Non-Toxin
	165	NLVNLILNSRWV	0.66	0.9442	No	Non-Toxin
	31	TGEALAALEAEI	0.63	0.5792	No	Non-Toxin
	172	NSRWVFQTTTSQ	0.59	0.7762	No	Non-Toxin
	37	ALEAEISSLMTI	0.57	0.5704	No	Non-Toxin
	110	LVNRDVSWVGSN	0.57	1.1391	No	Non-Toxin
	27	EAILTGEALAAL	0.53	0.3587	No	Non-Toxin
**VP 3**	127	NSRWVFQTTASQ	0.67	0.7402	No	Non-Toxin
	64	SLVNRDVSWVGS	0.64	1.2227	No	Non-Toxin
	108	SLSPTSRLQIQS	0.64	1.2747	No	Non-Toxin
	101	VGQNIFNSLSPT	0.63	0.4172	No	Non-Toxin
	23	SNFSLVASLVNQ	0.61	0.8688	No	Non-Toxin
	139	NQGLLSGEAILI	0.57	0.3629	No	Non-Toxin
	60	REQVSLVNRDVS	0.56	0.996	No	Non-Toxin
	18	TAEQFSNFSLVA	0.56	1.1533	No	Non-Toxin
	92	QWENSLLHSVGQ	0.52	0.0038	No	Non-Toxin
	31	LVNQGLTYGFIL	0.51	0.6342	No	Non-Toxin

Table S4. List of the top five vaccine models predicted by using the I-TASSER. The best one was selected based on C-score. This table presents the top five vaccine models generated using the I-TASSER protein structure prediction server. The I-TASSER algorithm utilizes a combination of threading, ab initio modeling, and structure assembly to predict the 3D structure of proteins. The vaccine models listed in the table were generated based on the primary amino acid sequence of the target antigen. The models are ranked based on their C-score, which is a confidence score provided by I-TASSER to estimate the quality of the predicted structure. A higher C-score indicates a higher confidence in the predicted model.

Model	C-score	Estimated TM-score	Estimated RMSD
**Model 1**	-1.37	0.55±0.15	10.9±4.6Å
**Model 2**	-1.48	N/o	N/o
**Model 3**	-4.00	N/o	N/o
**Model 4**	-2.59	N/o	N/o
**Model 5**	-4.19	N/o	N/o

Table S5. List of the predicted top five refined models from Galaxy refine server with different parameters for selection. This table presents the top five refined models generated using the Galaxy refine server with different parameters. The Galaxy refine server is a computational tool used for refining protein structure models to improve their quality and accuracy. Each refined model in the table was generated using a specific set of refinement parameters. These parameters may include optimization algorithms, energy functions, force fields, and other parameters that influence the refinement process.

Model	GDT-HA	RMSD	MolProbity	Clash score	Poor rotamers	Rama favored
**Initial**	1.0000	0.000	3.339	12.2	15.5	70.5
**MODEL 1**	0.9341	0.451	2.329	16.9	0.2	88.0
**MODEL 2**	0.9430	0.438	2.390	20.1	0.0	88.3
**MODEL 3**	0.9388	0.445	2.356	18.5	0.6	88.3
**MODEL 4**	0.9333	0.456	2.370	18.6	0.4	87.8
**MODEL 5**	0.9396	0.451	2.363	19.0	0.4	88.5

Table S6. Ramachandran plot score of initial and refined vaccine model and Z-score identified through ProSA-Web server. This table presents the Ramachandran plot analysis of both the initial and refined vaccine models, along with the corresponding Z-scores identified through the ProSA-Web server. The information presented in this table provides insights into the conformational quality and structural refinement of the vaccine models, as well as an assessment of their overall model quality based on the Z-scores obtained from the ProSA-Web server.

Conformations	Initial Model (Z-Score: -2.75)	Refine Model (Z-Score: -2.59)
**Highly Preferred**	428 (86.992%)	465 (94.512%)
**Preferred**	44 (8.943%)	24 (4.878%)
**Questionable**	20 (4.065%)	3 (0.610%)

Table S7. List of the energy scores for different conformations of vaccine-receptor complexes identified through ClusPro. This table presents the energy scores for different conformations of vaccine-receptor complexes obtained through the ClusPro protein-protein docking server. ClusPro is a widely used computational tool for predicting the structures and interactions of protein complexes. Each row in the table represents a different conformation of the vaccine-receptor complex, and the corresponding energy score is provided. The energy score reflects the stability and binding affinity of the complex, with lower scores indicating more favorable and stable interactions. 2.0.docking.

Cluster	Members	Representative	Weighted Score
0	44	Center	-996.1
		Lowest Energy	-1091.8
1	26	Center	-823.8
		Lowest Energy	-936.3
2	24	Center	-841.7
		Lowest Energy	-961.6
3	24	Center	-981.3
		Lowest Energy	-1006.5
4	18	Center	-972.5
		Lowest Energy	-972.5
5	18	Center	-925.9
		Lowest Energy	-925.9
6	17	Center	-829.2
		Lowest Energy	-989.8
7	16	Center	-1083.5
		Lowest Energy	-1122.9
8	14	Center	-982.6
		Lowest Energy	-982.6
9	14	Center	-978.6
		Lowest Energy	-1035.6

Table S8. List of amino acids interacted to form the vaccine-receptor complex. This table presents a list of amino acids that participate in the formation of the vaccine-receptor complex. The interactions between amino acids are described in terms of the residue involved, the closest neighboring residue, the distance between them, and the specific type of interaction observed.

Residue	Closest	Distance	Specific Interactions	# HB	# Salt Bridges	# Pi Stacking	# Disulfides	# vdW Clash	Surface Complementarity	Buried SASA
A:27:Glu	B:536:Tyr<br>B:540:Lys<br>B:539:Lys	2.6 A<br>2.6 A<br>2.9 A	1x hb to B:536:Tyr<br>1x hb to B:539:Lys<br>2x hb, 1x salt bridge, 1x clash to B:540:Lys	4	1	0	0	1	0.46	47.40%
A:28:Pro	B:536:Tyr<br>B:539:Lys<br>B:400:Ala	3.3 A<br>3.5 A<br>3.7 A	1x clash to B:536:Tyr	0	0	0	0	1	0.86	63.80%
A:29:Cys	B:539:Lys	3.5 A		0	0	0	0	0	0.6	100.00%
A:30:Val	B:399:Thr<br>B:400:Ala<br>B:543:Ile<br>B:397:Gly	3.4 A<br>3.6 A<br>3.8 A<br>4.0 A		0	0	0	0	0	0.74	100.00%
A:31:Glu	B:540:Lys<br>B:543:Ile	2.6 A<br>3.3 A	1x salt bridge, 4x clash to B:540:Lys<br>1x clash to B:543:Ile	0	1	0	0	5	0.6	81.00%

**A:32:Val**	B:396:Leu<br>B:547:Pro	3.7 A<br>3.8 A	1x clash to B:396:Leu	0	0	0	0	1	0.77	79.00%
**A:33:Val**				0	0	0	0	0	0	51.00%
**A:34:Pro**				0	0	0	0	0	0.61	56.70%
**A:39:Gln**	B:396:Leu<br>B:397:Gly	3.3 A<br>3.9 A	1x clash to B:396:Leu	0	0	0	0	1	0.82	50.30%
**A:41:Met**	B:395:Gln<br>B:397:Gly	3.8 A<br>4.0 A		0	0	0	0	0	0.82	62.70%
**A:42:Glu**	B:456:Leu<br>B:457:Ile<br>B:458:Leu	3.4 A<br>3.5 A<br>3.6 A		0	0	0	0	0	0.85	62.80%
**A:43:Leu**	B:458:Leu<br>B:457:Ile	3.3 A<br>3.9 A		0	0	0	0	0	0.84	93.40%
**A:44:Asn**	B:457:Ile<br>B:462:Trp	3.2 A<br>3.2 A	1x clash to B:457:Ile	0	0	0	0	1	0.85	86.20%
**A:45:Phe**				0	0	0	0	0	0	0.10%
**A:46:Tyr**				0	0	0	0	0	0	2.10%
**A:53:Pro**				0	0	0	0	0	0	0.10%
**A:63:Phe**	B:457:Ile	3.4 A		0	0	0	0	0	0.91	6.50%
**A:64:Asn**				0	0	0	0	0	0.89	0.00%
**A:65:Pro**				0	0	0	0	0	0.85	82.40%
**A:89:Glu**				0	0	0	0	0	0	0.80%
**A:450:Ile**	B:566:Lys	3.8 A		0	0	0	0	0	0.92	23.40%
**A:451:Tyr**				0	0	0	0	0	0	21.20%
**A:474:Glu**	B:566:Lys<br>B:567:Lys	2.5 A<br>3.1 A	1x hb, 1x salt bridge, 5x clash to B:566: Lys<br>1x hb to B:567:Lys	2	1	0	0	5	0.71	74.40%
**A:475:Val**	B:565:Pro<br>B:566:Lys	3.1 A<br>3.7 A		0	0	0	0	0	0.83	97.10%
**A:477:Lys**				0	0	0	0	0	0.32	17.70%
**A:499:Thr**	B:566:Lys<br>B:567:Lys	3.2 A<br>3.3 A	1x clash to B:567:Lys	0	0	0	0	1	0.83	97.70%
**A:500:Phe**	B:565:Pro<br>B:569:Phe	3.3 A<br>3.6 A	1x pi stack to B:569:Phe	0	0	1	0	0	0.77	74.40%
**A:505:Gln**				0	0	0	0	0	0	1.20%
**A:521:Ser**	B:567:Lys	3.8 A		0	0	0	0	0	0.75	30.90%
**A:523:Gln**	B:567:Lys<br>B:566:Lys<br>B:571:Ile<br>B:570:Ala	2.5 A<br>2.8 A<br>3.4 A<br>3.5 A	2x hb to B:567:Lys	2	0	0	0	0	0.65	100.00%
**A:524:Val**	B:569:Phe	3.5 A	1x clash to B:569:Phe	0	0	0	0	1	0.68	79.90%
**A:545:Ser**				0	0	0	0	0	0.71	28.60%
**A:547:Gln**	B:574:Glu<br>B:570:Ala<br>B:571:Ile	2.9 A<br>3.2 A<br>3.6 A	1x clash to B:570:Ala<br>2x clash to B:574:Glu	0	0	0	0	3	0.81	73.10%
**A:548:Val**				0	0	0	0	0	0.41	47.90%
**A:572:Ala**	B:574:Glu	3.2 A		0	0	0	0	0	0.8	57.20%
**A:573:Phe**	B:578:Leu<br>B:574:Glu	3.6 A<br>3.6 A	1x clash to B:574:Glu<br>1x clash to B:581:Lys	0	0	0	0	2	0.83	85.40%

	B:577:Asp<br>B:581:Lys	3.7 A<br>3.8 A								
**A:595:Lys**				0	0	0	0	0	0	4.00%
**A:598:Arg**	B:340:Asn	2.7 A	1x hb to B:340:Asn	1	0	0	0	0	0.13	36.40%
**A:599:Gln**				0	0	0	0	0	0.32	6.90%
**A:601:Leu**	B:581:Lys	2.6 A	1x hb to B:581:Lys	1	0	0	0	0	0.47	100.00%
**A:602:Val**	B:581:Lys	2.8 A	1x hb, 1x clash to B:581:Lys	1	0	0	0	1	0.76	40.10%
**A:603:Glu**	B:379:Arg	2.8 A	1x hb, 1x salt bridge to B:379:Arg	1	1	0	0	0	0	23.60%
**A:604:Val**	B:336:Met	3.3 A		0	0	0	0	0	0.43	83.10%
**A:605:Glu**	B:379:Arg	2.7 A	2x hb, 1x salt bridge, 1x clash to B:379:Arg	2	1	0	0	1	0.36	84.50%
**A:606:Arg**	B:379:Arg	3.9 A		0	0	0	0	0	0	13.00%
**A:619:Pro**				0	0	0	0	0	0.24	29.30%
**A:621:Leu**				0	0	0	0	0	0.24	55.90%
**A:622:Ser**	B:339:Leu<br>B:343:Trp	3.6 A<br>3.8 A		0	0	0	0	0	0.49	79.60%
**A:623:Leu**				0	0	0	0	0	0	4.10%
**A:624:Asn**				0	0	0	0	0	0	9.70%
**B:332:Pro**				0	0	0	0	0	0.51	24.70%
**B:335:Ile**				0	0	0	0	0	0.32	71.90%
**B:336:Met**	A:604:Val	3.3 A		0	0	0	0	0	0.43	63.30%
**B:339:Leu**	A:622:Ser	3.6 A		0	0	0	0	0	0.28	71.40%
**B:340:Asn**	A:598:Arg	2.7 A	1x hb to A:598:Arg	1	0	0	0	0	0	37.00%
**B:342:Leu**				0	0	0	0	0	0	0.90%
**B:343:Trp**	A:622:Ser	3.8 A		0	0	0	0	0	0.39	28.20%
**B:375:His**				0	0	0	0	0	0.68	14.10%
**B:379:Arg**	A:605:Glu<br>A:603:Glu<br>A:606:Arg	2.7 A<br>2.8 A<br>3.9 A	1x hb, 1x salt bridge to A:603:Glu<br>2x hb, 1x salt bridge, 1x clash to A:605:Glu	3	2	0	0	1	0.63	62.90%
**B:387:Lys**				0	0	0	0	0	0	0.40%
**B:395:Gln**	A:41:Met	3.8 A		0	0	0	0	0	0.84	12.40%
**B:396:Leu**	A:39:Gln<br>A:32:Val	3.3 A<br>3.7 A	1x clash to A:32:Val<br>1x clash to A:39:Gln	0	0	0	0	2	0.77	50.80%
**B:397:Gly**	A:39:Gln<br>A:30:Val<br>A:41:Met	3.9 A<br>4.0 A<br>4.0 A		0	0	0	0	0	0.85	90.70%
**B:398:Phe**				0	0	0	0	0	0.71	3.10%
**B:399:Thr**	A:30:Val	3.4 A		0	0	0	0	0	0.72	80.70%
**B:400:Ala**	A:30:Val<br>A:28:Pro	3.6 A<br>3.7 A		0	0	0	0	0	0.83	91.10%
**B:404:Ser**				0	0	0	0	0	0	22.90%
**B:447:Trp**				0	0	0	0	0	0	12.20%
**B:455:Asn**				0	0	0	0	0	0	0.40%
**B:456:Leu**	A:42:Glu	3.4 A		0	0	0	0	0	0.91	26.10%

**B:457:Ile**	A:44:Asn<br>A:63:Phe<br>A:42:Glu<br>A:43:Leu	3.2 A<br>3.4 A<br>3.5 A<br>3.9 A	1x clash to A:44:Asn	0	0	0	0	1	0.85	73.80%
**B:458:Leu**	A:43:Leu<br>A:42:Glu	3.3 A<br>3.6 A		0	0	0	0	0	0.84	82.50%
**B:461:Arg**				0	0	0	0	0	0	27.10%
**B:462:Trp**	A:44:Asn	3.2 A		0	0	0	0	0	0.79	32.80%
**B:465:Gln**				0	0	0	0	0	0	2.20%
**B:536:Tyr**	A:27:Glu<br>A:28:Pro	2.6 A<br>3.3 A	1x hb to A:27:Glu<br>1x clash to A:28:Pro	1	0	0	0	1	0.71	49.90%
**B:539:Lys**	A:27:Glu<br>A:28:Pro<br>A:29:Cys	2.9 A<br>3.5 A<br>3.5 A	1x hb to A:27:Glu	1	0	0	0	0	0.61	100.00%
**B:540:Lys**	A:27:Glu<br>A:31:Glu	2.6 A<br>2.6 A	2x hb, 1x salt bridge, 1x clash to A:27:Glu<br>1x salt bridge, 4x clash to A:31:Glu	2	2	0	0	5	0.5	71.20%
**B:543:Ile**	A:31:Glu<br>A:30:Val	3.3 A<br>3.8 A	1x clash to A:31:Glu	0	0	0	0	1	0.66	100.00%
**B:544:Gly**				0	0	0	0	0	0.53	82.20%
**B:547:Pro**	A:32:Val	3.8 A		0	0	0	0	0	0.35	68.20%
**B:548:Leu**				0	0	0	0	0	0.59	35.70%
**B:551:Gln**				0	0	0	0	0	0	20.80%
**B:561:Gln**				0	0	0	0	0	0	1.80%
**B:565:Pro**	A:475:Val<br>A:500:Phe	3.1 A<br>3.3 A		0	0	0	0	0	0.84	71.60%
**B:566:Lys**	A:474:Glu<br>A:523:Gln<br>A:499:Thr<br>A:475:Val<br>A:450:Ile	2.5 A<br>2.8 A<br>3.2 A<br>3.7 A<br>3.8 A	1x hb, 1x salt bridge, 5x clash to A:474:Glu	1	1	0	0	5	0.75	59.40%
**B:567:Lys**	A:523:Gln<br>A:474:Glu<br>A:499:Thr<br>A:521:Ser	2.5 A<br>3.1 A<br>3.3 A<br>3.8 A	1x hb to A:474:Glu<br>1x clash to A:499:Thr<br>2x hb to A:523:Gln	3	0	0	0	1	0.74	48.40%
**B:569:Phe**	A:524:Val<br>A:500:Phe	3.5 A<br>3.6 A	1x pi stack to A:500:Phe<br>1x clash to A:524:Val	0	0	1	0	1	0.7	73.90%
**B:570:Ala**	A:547:Gln<br>A:523:Gln	3.2 A<br>3.5 A	1x clash to A:547:Gln	0	0	0	0	1	0.8	100.00%
**B:571:Ile**	A:523:Gln<br>A:547:Gln	3.4 A<br>3.6 A		0	0	0	0	0	0.79	14.00%
**B:574:Glu**	A:547:Gln<br>A:572:Ala<br>A:573:Phe	2.9 A<br>3.2 A<br>3.6 A	2x clash to A:547:Gln<br>1x clash to A:573:Phe	0	0	0	0	3	0.75	90.10%
**B:575:Pro**				0	0	0	0	0	0	1.10%
**B:577:Asp**	A:573:Phe	3.7 A		0	0	0	0	0	0.9	38.80%
**B:578:Leu**	A:573:Phe	3.6 A		0	0	0	0	0	0.88	39.20%
**B:581:Lys**	A:601:Leu<br>A:602:Val<br>A:573:Phe	2.6 A<br>2.8 A<br>3.8 A	1x clash to A:573:Phe<br>1x hb to A:601:Leu	2	0	0	0	2	0.66	57.50%

			1x hb, 1x clash to A:602:Val							

Table S9. List of Cytotoxic T lymphocytes HLA (Human Leukocyte Antigen) alleles related to the epitope of interest. This table presents a list of HLA alleles that are associated with the cytotoxic T lymphocyte (CTL) epitope of interest. HLA alleles are proteins expressed on the surface of cells that play a critical role in presenting antigens to T cells and determining immune responses.

CTL Epitopes	HLA Allele
FELGCALDK	HLA-B*44:03; HLA-B*40:01; HLA-B*18:01 HLA-B*44:02; HLA-B*40:02
LNRKEREAL	HLA-B*14:02; HLA-B*08:0; HLA-B*07:02; HLA-B*48:01
LPFELGCAL	HLA-B*42:01; HLA-B*39:019; HLA-B*83:01; HLA-B*07:02 HLA-B*35:01; HLA-B*51:01; HLA-B*14:02; HLA-B*15:09 HLA-B*53:01; HLA-B*48:01; HLA-B*15:02; HLA-B*35:03; HLA-B*08:03 HLA-B*54:01; HLA-B*08:01; HLA-B*18:01; HLA-A*69:01
LVNRDVSWV	HLA-A*02:03; HLA-C*05:01; HLA-A*02:19; HLA-A*69:01 HLA-A*02:16; HLA-A*02:17; HLA-A*02:11; HLA-A*02:12; HLA-A*02:06 HLA-A*02:02; HLA-A*68:02
PRYFNVTLR	HLA-C*07:01; HLA-B*27:05; HLA-B*27:20
QLGFTAEQF	HLA-A*23:01; HLA-B*15:03; HLA-B*08:02; HLA-B*15:01; HLA-A*29:02 HLA-B*08:03
SVAPAAVTF	HLA-A*32:01; HLA-B*15:01; HLA-A*24:02; HLA-A*26:02 HLA-B*15:03; HLA-A*26:01; HLA-B*35:01; HLA-B*15:17; HLA-A*24:03 HLA-A*29:02; HLA-A*68:02; HLA-A*26:03; HLA-B*35:03; HLA-A*25:01 HLA-B*57:01; HLA-B*58:01; HLA-A*23:01; HLA-B*07:02 HLA-A*66:01; HLA-B*46:01; HLA-B*15:02; HLA-B*58:02; HLA-A*32:15 HLA-C*07:02
TLEETDYCL	HLA-A*02:12; HLA-A*02:19; HLA-B*39:01; HLA-C*05:01; HLA-A*02:02 HLA-B*15:02; HLA-A*02:11; HLA-A*02:16; HLA-A*02:17; HLA-B*38:01 HLA-A*02:01

Table S10. List of Helper T lymphocytes HLA (Human Leukocyte Antigen) alleles related to the epitope of interest. This table provides a comprehensive list of HLA alleles associated with the epitope of interest specifically in relation to Helper T lymphocytes. HLA alleles play a crucial role in the immune system by presenting antigens to Helper T cells, which then help coordinate and regulate immune responses.

HTL Epitopes	HLA Allele
AQLGFTAEQFSNFSL	HLA-DPA1*02:01/DPB1*05:01; HLA-DPA1*01:03/DPB1*02:01
CDTLQMWEAISVKTE	HLA-DRB1*04:02; HLA-DQA1*01:01/DQB1*05:01; HLA-DRB1*15:01 HLA-DRB1*15:06; HLA-DQA1*03:01/DQB1*03:02
CFCYQCFILWFGFPP	HLA-DQA1*01:01/DQB1*05:01; HLA-DPA1*01:03/DPB1*02:01
FKVDFKSRHACELGC	HLA-DRB1*03:05; HLA-DRB1*03:09; HLA-DRB1*11:07; HLA-DRB1*03:06 HLA-DRB1*03:07; HLA-DRB1*03:08; HLA-DRB1*08:01
FNVTLRKRWVKNPYP	HLA-DRB1*08:04; HLA-DRB1*08:06; HLA-DRB1*11:01
RHALMAFSLDPLQWE	HLA-DRB4*01:01; HLA-DQA1*01:01/DQB1*05:01; HLA-DQA1*05:01/DQB1*02:01 HLA-DPA1*03:01/DPB1*04:02; HLA-DPA1*02:01/DPB1*01:01

VIMMELNTLWSKFQQ	HLA-DRB1*11:04; HLA-DRB1*11:06; HLA-DRB1*13:11; HLA-DRB1*11:04<br>HLA-DRB1*11:06; HLA-DRB1*13:11; HLA-DRB1*04:23; HLA-DRB1*04:23<br>HLA-DRB1*04:04, HLA-DRB1*04:04; HLA-DRB1*15:06; HLA-DRB1*15:06<br>HLA-DRB5*01:05; HLA-DRB5*01:05; HLA-DRB1*04:08; HLA-DRB1*04:08<br>HLA-DRB1*04:01; HLA-DRB1*04:26; HLA-DRB1*04:01; HLA-DRB1*04:26<br>HLA-DRB1*15:02; HLA-DRB1*15:02; HLA-DRB1*04:10; HLA-DRB1*04:10
VNLILNSRWVFQTTA	HLA-DRB1*04:10; HLA-DRB1*13:01; HLA-DRB1*13:27; HLA-DRB1*13:28<br>HLA-DRB1*04:23; HLA-DRB1*11:20; HLA-DRB1*04:08; HLA-DRB1*03:01
